# Brr2p carboxy-terminal Sec63 domain modulates Prp16 splicing RNA helicase

**DOI:** 10.1093/nar/gku1238

**Published:** 2014-11-26

**Authors:** Olivier Cordin, Daniela Hahn, Ross Alexander, Amit Gautam, Cosmin Saveanu, J. David Barrass, Jean D. Beggs

**Affiliations:** 1 Wellcome Trust Centre for Cell Biology, University of Edinburgh, King's Buildings, Mayfield Road, Edinburgh, EH9 3BF, UK; 2 IBPC, CNRS FRE 3630, 13, rue Pierre & Marie Curie, 75005 Paris, France; 3 Institut Pasteur, CNRS UMR3525, 25-28 rue du docteur Roux, 75015 Paris, France

## Abstract

RNA helicases are essential for virtually all cellular processes, however, their regulation is poorly understood. The activities of eight RNA helicases are required for pre-mRNA splicing. Amongst these, Brr2p is unusual in having two helicase modules, of which only the amino-terminal helicase domain appears to be catalytically active. Using genetic and biochemical approaches, we investigated interaction of the carboxy-terminal helicase module, in particular the carboxy-terminal Sec63-2 domain, with the splicing RNA helicase Prp16p. Combining mutations in *BRR2* and *PRP16* suppresses or enhances physical interaction and growth defects in an allele-specific manner, signifying functional interactions. Notably, we show that Brr2p Sec63-2 domain can modulate the ATPase activity of Prp16p *in vitro* by interfering with its ability to bind RNA. We therefore propose that the carboxy-terminal helicase module of Brr2p acquired a regulatory function that allows Brr2p to modulate the ATPase activity of Prp16p in the spliceosome by controlling access to its RNA substrate/cofactor.

## INTRODUCTION

Nuclear pre-mRNA splicing is catalyzed by the spliceosome complex, composed of five small nuclear ribonucleoprotein particles (snRNPs; U1, U2, U4, U5 and U6) and a large number of non-snRNP proteins (1). Spliceosome assembly is a multi-step process and the assembled spliceosome is also highly dynamic due to conformational rearrangements (1). Eight DExD/H RNA helicases participate in pre-mRNA splicing, and have been proposed to remodel RNA–protein or RNA–RNA interactions within splicing complexes (1–3). DExD/H helicases are RNA-dependent molecular motors that derive energy from adenosine triphosphate (ATP) hydrolysis (4).

Brr2p may have multiple roles in pre-mRNA splicing. Prior to the first catalytic step, Brr2p disrupts the U4/U6 snRNA duplex, an essential step in formation of the catalytic core of the spliceosome (5). Prior to the second catalytic step, it apparently helps to position the 3′ splice site of introns that have highly structured 3′ ends (6), and it was proposed to function also in spliceosome disassembly (7). However, the latter two roles seem to be independent of Brr2′s ATPase activity (6,8,9). Brr2p is a core component of the mature U5 snRNP and remains associated with the spliceosome throughout the splicing cycle, interacting with the key splicing factors Snu114p and Prp8p (10,11). Snu114p and Prp8p are thought to control intermittent stimulation and repression of Brr2p activity at different stages of the splicing cycle (7,9,12–16). Notably, the Jab1 domain of Prp8p was recently shown to interact with the RNA binding cleft in the amino terminal helicase domain of Brr2p *in vitro*, inhibiting RNA binding there (15,16).

**Figure 1.**
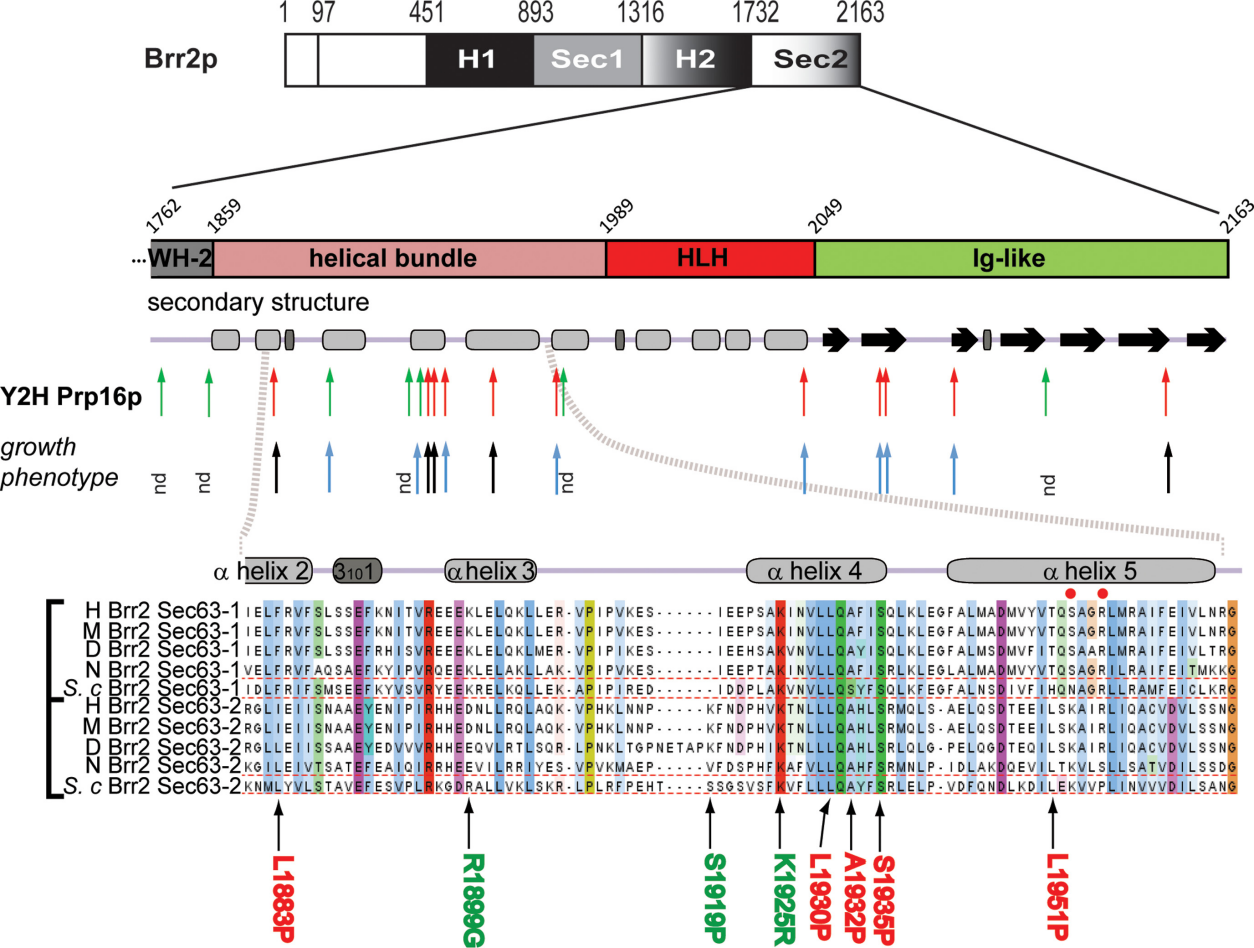
Identification and phenotypic characterization of mutations in the C-terminal domains of Brr2p that alter interaction with Prp16p. Overview of single amino-acid substitutions within the Brr2p WH-Sec63-2 domains identified by a genetic screen for alleles with perturbed protein–protein interactions, and schematic of secondary structure elements. Arrows indicate where mutations cause weaker (red) or stronger (green) Y2H interactions with Prp16p and heat-sensitive (black) or normal (blue) growth. (Bottom) Multiple sequence alignment of the indicated region within the Sec63-1 and Sec63-2 domains of Brr2p. H—human; M—mouse; D—*Drosophila melanogaster*; N—*Neurospora crassa*; *S. c*.—*Saccharomyces cerevisiae*. Bold color indicates conservation. Red dots in helix α5 indicate residues in the Sec63-1 domain known to affect Brr2p function (7,19).

Structural analyses have shown that Brr2p has two consecutive helicase modules, each consisting of a Ski-2 like helicase (H) domain and a Sec63 domain, separated by a winged helix (WH) domain (Figure 1A) (17–19). This domain organization of Brr2p is highly conserved in eukaryotes. Only the conserved N-terminal helicase module, referred to as H1-Sec63-1, displays catalytic activity (20). In contrast to the N-terminal domain, the C-terminal helicase and Sec63 domains (H2 and Sec63-2) are relatively degenerate in sequence, and mutations within ATPase motifs I and II of H2 do not cause growth defects or loss of activity (20). However, deletion of the entire C-terminal module (hereafter referred to as H2-Sec63-2) is lethal in budding yeast. Deleting only the Sec63-2 domain strongly affects cell growth (19), indicating that, although catalytically inactive, this domain has an important function. Indeed, it was recently reported that the C-terminal region of Brr2p modulates the activity of the N-terminal helicase module (18).

In a yeast two-hybrid (Y2H) screen, the C-terminal part of budding yeast Brr2p was found to interact with the spliceosomal helicases Prp2p and Prp16p and with other splicing factors, possibly as a protein interaction platform (21,22). It has been proposed that Brr2p functions as a receptor for Prp16p (21) and Prp2p (23) in the spliceosome.

Here we present a combination of *in vivo* and *in vitro* approaches to further characterize the interaction of Brr2p C-terminus with Prp16p. First, we identified several mutations in Brr2p Sec63-2p that alter the interaction with Prp2p and/or Prp16p. Notably, these cluster in a particular structural element in the Sec63-2 domain. Focusing on the relationship between Brr2p C-terminus and Prp16p, we find that certain combinations of *prp16* and *brr2* mutant alleles result in synthetic growth phenotypes, supporting the functional significance of the physical interactions. We show that Prp16p interacts directly with the Sec63-2 domain of Brr2p. This interaction interferes with RNA binding to Prp16p and the RNA stimulation of ATP hydrolysis by Prp16p. We therefore propose that Brr2p Sec63-2 domain could participate in regulating the activation of Prp16p at the catalytic center of the spliceosome by controlling access to its RNA substrate/cofactor.

## MATERIALS AND METHODS

Plasmids and strains are listed in Supplementary Tables S1 and S2, respectively.

### Cloning procedures

Brr2p domains H2-Sec63-2 and Sec63-2 were polymerase chain reaction (PCR)-amplified and cloned into pBlueScript using SpeI/XhoI restriction sites. LexA-bait fusions of *PRP2* and *PRP16* were generated by InFusion cloning (Takara). The Gal4AD-prey fusion for H2-Sec63-2p was constructed in pACTII-stop using XmaI/BamHI sites. Mutations in *PRP16* were created by site-directed mutagenesis (Quick Change, Promega).

### Yeast two-hybrid assay

Y2H assays were performed using the haploid L40ΔG yeast strain co-transformed with LexA-bait and Gal4AD-prey plasmids, selecting for expression of the *HIS3* reporter gene (growth on medium lacking leucine and tryptophan (-LW) to select for both plasmids and lacking histidine (-H) to select for *HIS3* expression; -LWH medium) (21,24) and including various concentrations of 3-aminotriazole (3-AT) that increases the stringency of the *HIS3* activation, allowing an assessment of the strength of interaction. Western blotting of bait and prey-fusions using anti-HA (F-7) and anti-LexA (2–12) antibodies (Santa Cruz Biotechnology) showed stable expression of the fusion proteins, regardless of the Y2H interaction phenotype observed.

### BRR2 H2-Sec63-2 mutant library construction

Amino-acids 1762 to 2163 of Brr2p were randomly mutated by error-prone PCR using non-proofreading TaKaRa LA TaqTM polymerase with 0.75-mM MnCl_2_, then integrated into pACTII-stop plasmid by Megaprimer PCR. *DpnI*-treated PCR products were transformed to *Escherichia coli* XL10 Gold (Stratagene). Plasmids from 50 clones were sequenced to determine the mutation-frequency prior to pooling the transformants.

### Identification of mutants with aberrant protein interactions

Yeast L40ΔG cells were transformed with a LexA-bait fusion (Prp2p or Prp16p) and the library of mutated H2-Sec63-2 plasmids. A Singer ROTOR yeast handling robot spotted stationary phase cultures of single yeast clones in 384-sample format on agar plates. After 2 days at 30°C, clones were replica-plated onto selective medium (-LWH), supplemented with different concentrations of 3-AT, and incubated at 30 or 14°C before comparison of colony size with wild-type (WT). Clones with a ‘stronger’ Y2H interaction were able to grow on medium containing 3-AT concentrations (up to 65 mM for Prp16p; up to 20 mM for Prp2p) that were toxic with WT H2-Sec63. Clones with a ‘weaker’ interaction grew on selective medium (minus histidine) at 14°C but not at 30°C and/or showed reduced or no resistance to 3-AT. Sequencing H2-Sec63-2 prey constructs identified the mutations in selected mutants. To confirm the observed phenotypes, prey plasmids were recovered, re-transformed in the Y2H strains and the interaction with the original baits or other bait constructs assessed.

### Plasmid shuffle and growth assay

A W303 *brr2Δ* strain was constructed by replacing the entire open reading frame (ORF) from one chromosomal copy of *BRR2* with the kanMX6 cassette in W303 diploid cells (25), transformation with pRS316-*BRR2* (*URA3*), sporulation and isolation of *brr2Δ* haploids carrying pRS316-*BRR2*. Mutant derivatives of pRS315-*BRR2* were generated by site-directed mutagenesis (Quick Change, Promega) and their phenotypes analyzed after plasmid shuffle on 5-FOA to select for clones that had lost pRS316-*BRR2* (26). 5-FOA-selected transformants were spotted on YPDA (yeast peptone, dextrose, adenine; complete, non-selective medium) agar and incubated for 2 days at 25°, 30°, 37°C or for 5 days at 18° or 15°C. W303 *brr2Δ/isy1Δ* was constructed by replacing the entire *ISY1* ORF with natNT2 (27). W303 *brr2Δ/prp16Δ* was constructed by replacing one genomic copy of the entire *PRP16* ORF with natNT2 in a diploid, heterozygous *brr2*Δ strain carrying pRS316-*BRR2/PRP16*, followed by sporulation and tetrad dissection. Mutant derivatives of a pRS314-*PRP16* plasmid were constructed by site-directed mutagenesis. W303 *brr2Δ/prp16Δ* was co-transformed with pRS314-*PRP16* and pRS315-*BRR2* or mutant versions thereof for plasmid shuffle assays on 5-FOA to lose pRS316-*BRR2/PRP16*.

### RT-qPCR

Total RNA was purified and RT-qPCR performed as in (28). cDNA was prepared from 5μg of DNase-treated RNA in a 10-μl reaction mixture containing 5x First strand synthesis buffer, 0.1-M dithiothreitol (DTT), 10-U RNase inhibitor (Roche), 10 mM of each deoxyribonucleotide, 250 nM mix of gene-specific primers and 7.5-U Transcriptor (Roche). Residual RNA was hydrolyzed by the addition of 15 μl of 0.1-mg/ml RNaseA and incubation at 37°C for 1 h. cDNA was then diluted 1/20. qPCRs were performed in triplicate with Brilliant III SYBR Master mix (Agilent) in a Roche LC480. Reaction volumes were 10 μl, (4μl 2x SYBR green qPCR mix and 300 nM each primer, 4μl of cDNA template). Cycling parameters were: 3 min at 94°C, then 50 cycles of 5 s at 94°C, 10 s at 60°C. Oligonucleotides are listed in Supplementary Table S3. Figure 3A illustrates positions of amplicons.

**Figure 2.**
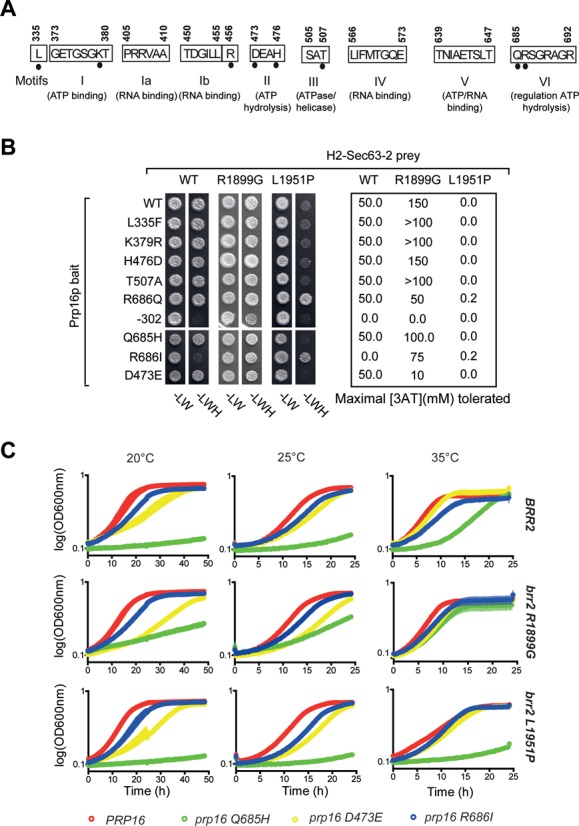
Mutations in the catalytic core of Prp16p influence its Y2H interaction with the C-terminus of Brr2p and confer synthetic growth defects with certain *brr2* mutant alleles. (**A**) Schematic showing the conserved motifs of the Prp16p helicase core. Black dots indicate residues substituted in different *prp16* mutants. (**B**) Y2H interaction assay of Prp16 mutant proteins and Brr2 H2-Sec63-2p. Numbers to the right indicate the highest concentration [mM] of 3-AT tolerated. (**C**) Growth phenotypes of *PRP16* (red line), *prp16-D473E* (yellow line), *prp16-Q685H* (green line) and *prp16-R686I* (black line), in combination with *BRR2*, *brr2-R1899G* or *brr2-L1951P*. Cells were grown in liquid -L-W medium at 20, 25 or 35°C. Data were recorded for four independent cultures; line thickness reflects the standard error to the mean.

**Figure 3.**
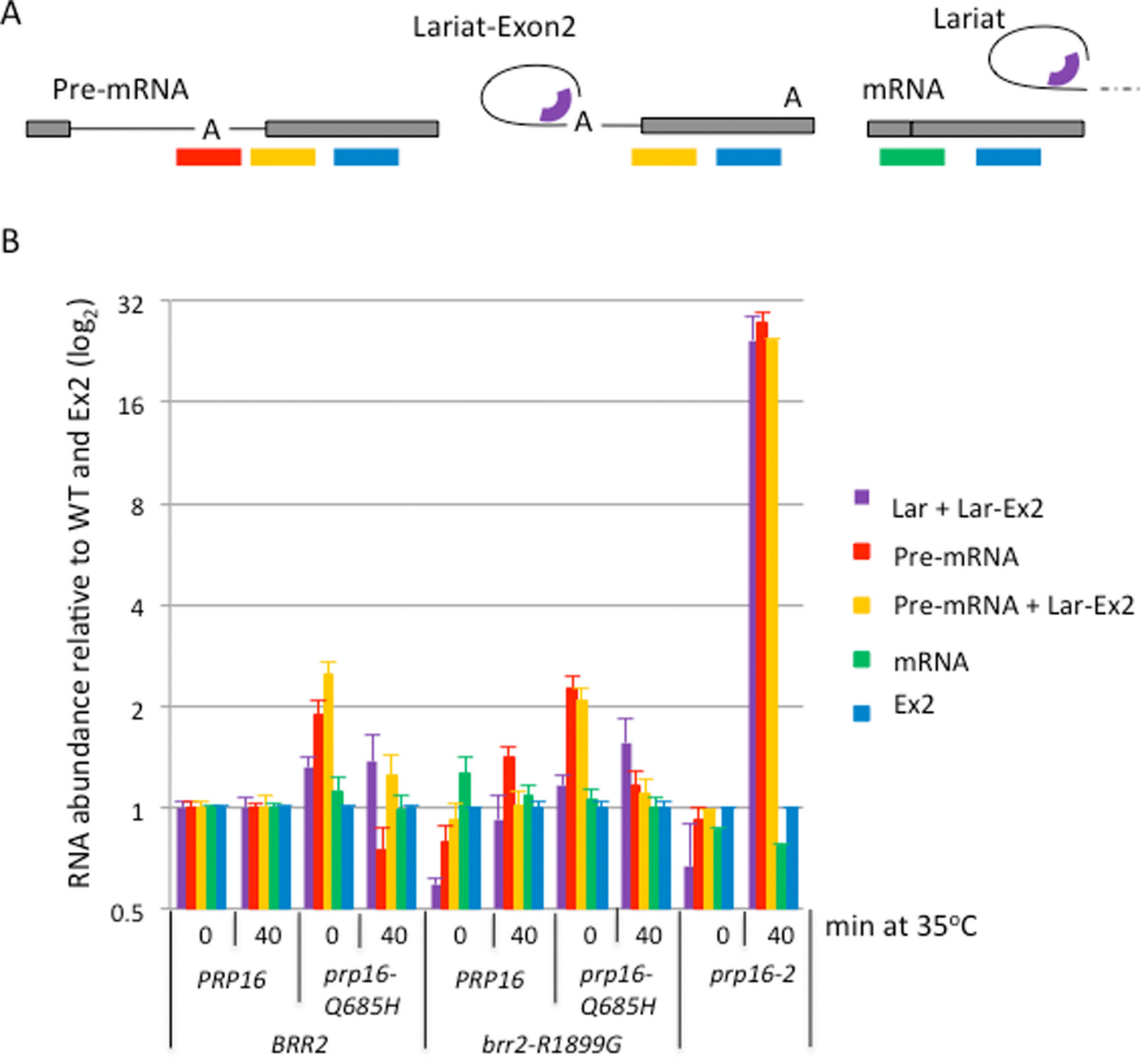
Splicing analysis for *prp16-Q685H* and *brr2-R1899G*. (**A**) Positions of the qPCR amplicons used to measure across the linear branch point region (detects only pre-mRNA), branched structure (measures lariat-exon2 and excised lariat), 3′ splice site (measures lariat-exon2 and pre-mRNA), spliced exons (measures mRNA) and exon2 (measures all species) of *ACT1* transcripts. (**B**) Results (log base 2, normalized to WT and exon2 levels) are shown for the indicated strains grown at 25°C or after shifting to 35°C for 40 min. Error bars indicate SEM for three biological replicates and three technical replicates.

### Protein production and purification

All proteins were produced in *E. coli* BL21 RIL (Stratagene), using plasmids described in Supplementary Table S1. Cultures were grown in LB with ampicillin (50 μg/ml) and chloramphenicol (30 μg/ml) at 30°C to OD_600_ 0.6, then transferred to 18°C for 1 h before addition of isopropyl β-D-1-thiogalactopyranoside (IPTG) to 0.4 mM and incubation overnight at 18°C. Cell pellets were resuspended in 25-ml lysis buffer (50-mM Tris–HCl pH 7.5, 500-mM NaCl, 20-mM imidazole, 1-mM DTT, 2-mM 2-mercaptoethanol, 10% glycerol). After incubation with egg-white Lysozyme on ice for 30 min, lysates were sonicated (VXC400, Jencons Scientific ltd), centrifuged for 30 min at 17 000 rpm (Beckman Avanti J-25) then filtered using a 0.22 μm syringe filter and loaded on a Hi-Trap IMAC HP (GE healthcare) charged with NiCl. The column was washed with 20 volumes of lysis buffer, and the target proteins were eluted using a linear gradient of imidazole (10 to 500 mM) in lysis buffer. After desalting, buffer exchange (50-mM Tris–HCl pH 7.5, 100-mM NaCl, 10-mM imidazole, 1-mM DTT) and concentration, glycerol concentration was adjusted to 50% and the purified protein snap frozen in liquid nitrogen. Prp16p was purified using NiNTA-agarose resin (Qiagen) following the manufacturer's recommendations. Ded1p and Prp22p were purified as previously described (29,30).

### Co-immunoprecipitation assay

Prp16p (3 μg) and Sec63-2p (1.5 μg) were incubated overnight at 4°C with protein A dynabeads (Roche) and anti-Prp16p-029 (Eurogentec, this study) at 1/100 dilution in IP buffer (50-mM Tris–HCl, pH 7.5, 150-mM NaCl, 1-mM MgSO_4_, 0.02% (v/v) NP-40) containing bovine serum albumin (BSA) (100 μg/ml), glycogen (50 μg/ml) and tRNA (100 μg/ml). For co-immunoprecipitations treated with RNase A (30 ng/μl), tRNA was omitted. After extensive washing with IP buffer, beads were loaded onto NuPAGE 4–12% gradient gels (Invitrogen), followed by western blotting using anti-His-HRP-conjugated antibodies (sc-8036, Santa Cruz; 1/3000 dilution).

### Electrophoresis mobility shift assays (EMSA)

A 44-nt RNA (5′ GGG CGA AUU CAA AAC AAA ACA AAA CUA GCA CCG UAA AGC AAG CU 3′) was produced by *in vitro* transcription using the megashortscript kit (Ambion), purified and labeled as in (29). Electrophoresis mobility shift assays (EMSA) were performed as in (29) using the same buffer as for Prp16p ATPase assays (but excluding ATP), and 2-nM ^32^P 5′end-labeled RNA. Signals were detected by phosphoimaging (Fujifilm FLA-5100) and analyzed using AIDA software (Raytest). To account for the background, RNA bound was calculated as (bound/total lane) ×100.

### ATPase assay

ATP hydrolysis was measured as in (29) with minor modifications. Prp16p and Prp22p assays contained 20-mM Tris–HCl pH 7.5, 75-mM KCl, 1-mM MgSO_4_, 1-mM DTT, 0.1-μg/μl BSA, 1-mM ATP, 50-ng/μl total yeast RNA and enzyme at 10, 20 and 10 nM respectively. Ded1p assays contained 5-nM enzyme, 20-mM Tris–HCl pH 7.5, 50-mM KAc, 1-mM MgOAc, 0.1-μg/μl BSA, 2-mM DTT, 1-mM ATP and 50-ng/μl total yeast RNA. Incubations were conducted at 30°C for up to 45 min. Results were converted to ‘hydrolyzed Pi’ using a Pi standard curve specific for each experiment and plotted using GraphPad Prism 5.0.

## RESULTS

### Identification of mutant Brr2 proteins that have abnormal interactions with Prp2p or Prp16p

Based on Y2H and immunoprecipitation results that revealed interactions of Brr2p with Prp2p and Prp16p, we previously proposed that the C-terminus of Brr2p could act as a receptor for Prp16p, and possibly other DEAH-box helicases, at the catalytic center of the spliceosome (21). To investigate this further, we generated a library of H2-Sec63-2 Y2H variants in which part of WH2 (second winged-helix domain) and all of Sec63-2 (aa1762–2163) were randomly mutated by error-prone PCR, incorporating an average of 1.7 coding mutations per construct. We then searched amongst these for *brr2* mutations that reduce or enhance interaction with either Prp16p or Prp2p in two independent Y2H screens (see ‘Materials and Methods’ section). From ∼4000 clones that were tested, 272 were selected that showed altered growth with different concentrations of 3-aminotriazole (3-AT, which allows monitoring the strength of interactions *in vivo*) at 14, 30 or 37°C. These were sequenced and 18 *brr2* H2-Sec63-2 domain mutants that have only a single amino acid substitution were selected for further characterization (Figure 1 and Table 1). The mutants were characterized as exhibiting a stronger Y2H interaction based on the ability to grow in the presence of higher concentrations of 3-AT compared to WT, or a weaker Y2H interaction if they were only able to grow in the absence of histidine at reduced temperature and/or showed increased sensitivity to 3-AT (Supplementary Figure S1). Whether originally identified as having altered interaction with Prp2p or with Prp16p, most of the mutations affected interaction with both these proteins, and the interaction phenotype (e.g. stronger or weaker Y2H interaction) was generally conserved (Table 1). With the few mutations that affected only the interaction with either Prp2p or Prp16p, the effects were subtle. All mutants tested showed stable expression, similar to WT, at various temperatures, regardless of the Y2H interaction observed (data not shown).

**Table 1.** *brr2* alleles with aberrant Y2H protein–protein interaction phenotype

*brr2* allele^a^	Origin of allele	Y2H phenotype^b^ with		Growth phenotype^c^
		Prp2p	Prp16p	
single aa substitution, stronger Y2H interaction				
H1855R	screen with Prp16 as bait	WT	stronger	Nd
R1899G		stronger	stronger	WT
S1919P		weaker	stronger	Nd
K1925R		WT	stronger	WT
C1769R	screen with Prp2 as bait	stronger	stronger	WT
A1973N		stronger	stronger	Nd
L2120V		stronger	stronger	Nd

single aa substitution, weaker Y2H interaction				
L1883P	screen with Prp16bait	weaker	weaker	Ts
L1930P		weaker	weaker	Ts
A1932P		weaker	weaker	Ts
S1935P		weaker	weaker	WT
L1951P		weaker	weaker	Ts
N1972D		weaker	weaker	WT
V2045D		weaker	weaker	WT
I2071T		weaker	weaker	WT
I2073N		weaker	weaker	Nd
W2099R		weaker	weaker	slow growth at 37°C
S2148P		weaker	weaker	Ts

Y2H, Yeast Two-Hybrid; aa, amino acid; WT, wild-type; Ts, temperature sensitive; Nd, not determined.

^a^Identified aa substitutions, the given positions correspond to full length Brr2p.

^b^Strength of interaction as compared to WT H2-Sec63-2p (see Material and Methods). Stronger interactions were identified based on increased resistance to 3-AT. Weaker interactions were identified based on heat sensitivity and/or decreased resistance to 3-AT.

^c^Plasmid shuffle and growth assay of indicated aa substitutions introduced to full length *BRR2*. Serial dilutions of 5-FOA selected cells were spotted to YPDA agar and incubated at 16, 18, 25, 30 or 37°C.

The Brr2p Sec63-2 domain comprises three structural sub-domains (Figure 1, and Supplementary Figure S2A) that contact each other (19). Amongst the 18 single mutations that cause aberrant Y2H interactions, only two were located in the WH-2 domain whereas 10 mapped in the helical bundle domain (residues 1859–1989), clustering in or around helices α3, α4 and α5 (Figure 1 and Supplementary Figure S2A). The remaining mutations were located in the Fibronectin (IgG)-like domain. Multiple sequence alignments reveal a high level of conservation of the affected residues (Figure 1, bottom), indicating a functional significance of this region of the Sec63-2 domain. In the crystal structures of both yeast and human Brr2p, the Sec63-2 domain does not interact with the amino terminal helicase module (Supplementary Figure S2A) (15,16).

### ‘Weaker interaction’ mutations in Sec63-2 confer heat sensitivity and splicing defects

To address whether mutations in Sec63-2 affect the function of Brr2p *in vivo*, we transferred various substitutions into full-length *BRR2* and tested their effects on growth. Alleles that conferred stronger interactions in the Y2H assay did not cause growth defects (Table 1). Five alleles conferred heat-sensitive (ts) growth at 37°C (Supplementary Figure S2B). All of these ts mutants caused a weakened Y2H interaction with Prp16p (Table 1) and had acquired a proline substitution. This type of mutation may destabilize the structure of this domain at elevated temperature, but all were viable at 30°C. Indeed, *brr2*-*L1951P* cells grown at permissive temperature showed little or no splicing defect but accumulated unspliced RNA when incubated at 37°C (Supplementary Figure S2C). These results indicate that the second Sec63 domain of Brr2p is important for Brr2p activity *in vivo* and that some structural elements, such as helix α5, are necessary for Brr2p stability and/or function.

A Genetic Interaction Mapping (GIM) screen (31) to identify mutations that confer synthetic sickness in combination with *brr2-L1930P* or *brr2*-*L1951P* at 30°C identified several splicing factor mutations as genetic interactors. Amongst those, we identified *isy1Δ* as one of the strongest interactors, being synthetic lethal with both *brr2* alleles when tested at 37°C (Supplementary Figure S3 and Table S4). Significantly, *ISY1* also has a close genetic interaction with *PRP16.* Deletion of *ISY1* rescues the growth defect caused by the allele *prp16-302* and alleviates the stalling of spliceosomes that contain Prp16-302p (32).

### Mutations in the helicase core of Prp16p affect its interaction with Brr2p C-terminus

To better understand the relationship between Brr2p C-terminus and Prp16p functions *in vivo*, we first tested the effects of previously characterized mutations in the active site of Prp16p (33–37) (Figure 2A) on its interaction with the Brr2p C-terminus, using pair-wise Y2H tests. Most of the *prp16* mutations did not modify the Y2H interaction with H2-Sec63-2p (Figure 2B). Notable exceptions were *prp16*-*R686I* (motif VI) and *prp16-302* (contains two mutations, R456K next to motif Ib and G691R in motif VI), that strongly reduced interaction with H2-Sec63-2p. However, *brr2 H2-Sec63-2 R1899G*, originally isolated as a stronger interactor of Prp16p, rescued the defective interaction with the mutant Prp16-R686I protein and partially rescued the interaction with the Prp16-302 protein (in absence of 3-AT; Figure 2B). Curiously, *H2-Sec63-2 L1951P*, that has acquired a proline substitution and which was isolated as a weaker interactor, showed residual interaction exclusively with *prp16* motif VI mutants R686Q and R686I (Figure 2B). In the case of *prp16-Q685H*, the Y2H interaction was abolished by *brr2-L1951P* but enhanced by *brr2-R1899G.* These allele-specific effects indicate that the mutations identified in *brr2 Sec63-2* during the Y2H screen did not alter the interactions with Prp16p simply through non-specific effects on Brr2p structure. It appears that mutations in the catalytic core of Prp16p, particularly in the conserved helicase VI motif, can reduce its capacity to bind to the C-terminus of Brr2p, but the defect is suppressed by certain mutations in the Brr2p Sec63-2 domain.

### brr2 Sec63-2 alleles interact genetically with mutations in PRP16

Having studied the interactions of Brr2p C-terminus and Prp16p in the context of Y2H experiments, we addressed the link between Brr2p C-terminus and Prp16p activity *in vivo*. We used the plasmid shuffle approach to obtain strains carrying different combinations of *brr2* and *prp16* mutant alleles (33–34) as the only copies of these genes, and investigated their effects on growth at various temperatures when expressed as full-length proteins from their own promoters (Figure 2C). Of the two *brr2* mutants tested, *brr2-R1899G* showed no growth defect whereas *brr2-L1951P* caused a heat-sensitive growth defect in the WT *PRP16* background (Figure 2C, red lines). As previously reported (33), *prp16*-*D473E* caused slow growth at 20°C in the presence of WT *BRR2* (Figure 2C, yellow lines). This defect was enhanced in combination with *brr2*-*R1899G* or *brr2-L1951P* (which both reduced the Y2H interaction). In the *BRR2* background, *prp16*-*Q685H* caused very poor growth at all temperatures tested, the best growth being at 35°C (Figure 2C, green lines). This phenotype was exacerbated when combined with *brr2-L1951P* (which greatly reduced the Y2H interaction) but, strikingly, growth was improved in the presence of *brr2*-*R1899G* (which improved the Y2H interaction). The *prp16*-*R686I* allele (Figure 2C, blue lines) caused only a mild growth defect but was more similar to WT *PRP16* when combined with the *brr2-L1951P* mutation at 35°C, compatible with their improved Y2H interaction. Thus, mutations in the C-terminal region of *BRR2* show allele-specific interactions with mutations in the active site of *PRP16* that, to a large extent, mirror the effects on the Y2H interactions.

### The prp16 mutants display only very mild splicing defects for several intron-containing genes

Typically, *prp16* mutations cause a second step splicing defect (35). However, initial RT-qPCR analysis of the *prp16-D473E, prp16*-*Q685H* and *prp16-R686I* mutants detected only very low levels of either lariat or pre-mRNA accumulation for *ACT1* at 25 or 35°C (data not shown). As the most striking effect on growth was seen on combining the *brr2-R1889G* and *prp16-Q685H* mutations, we analyzed splicing of several intron-containing transcripts by RT-qPCR in the single and double mutant strains as well as in the well characterized *prp16-2* mutant. Figure 3 shows the levels of pre-mRNA, excised lariat/lariat-exon2, mRNA and exon2 for the *ACT1* intron-containing gene in cultures grown at 25°C and 40 min after shifting to 35°C. The *prp16-2* strain showed no pre-mRNA or lariat accumulation relative to WT at 25°C but accumulated high levels of lariat-exon2 after shifting to 35°C, with pre-mRNA also building up by 40 min (Figure 3B and data not shown). This can be explained by a defective second step of splicing that feeds back on the first step as availability of splicing factors becomes limiting due to stalled splicing complexes. In contrast, the *prp16-Q685H* mutant produced a low level accumulation of pre-mRNA, indicating a mild first step splicing defect at 25°C that switched to a very mild second step defect at 35°C, with only 5% the amount of lariat seen in the *prp16-*2 mutant. This mild splicing defect seems insufficient to cause the severe growth defect caused by the *prp16-Q685H* allele. The *brr2-R1899G* mutation displayed a mild first step defect at 35°C. At 25°C the *brr2-R1889G, prp16-Q685H* double mutant retained the first step defect of the *prp16-Q685H* allele. At 35°C the double mutant displayed only a low level of lariat accumulation, which may correspond to excised intron, due to the absence of lariat-exon2 signal (RT-qPCR across the 3′SS). Similar analyses of the intron-containing *DBP2* and *RPL28* transcripts showed only a similarly mild or no splicing defect (data not shown).

### Brr2 Sec63-2p interacts directly with Prp16p*in vitro*

**Figure 4.**
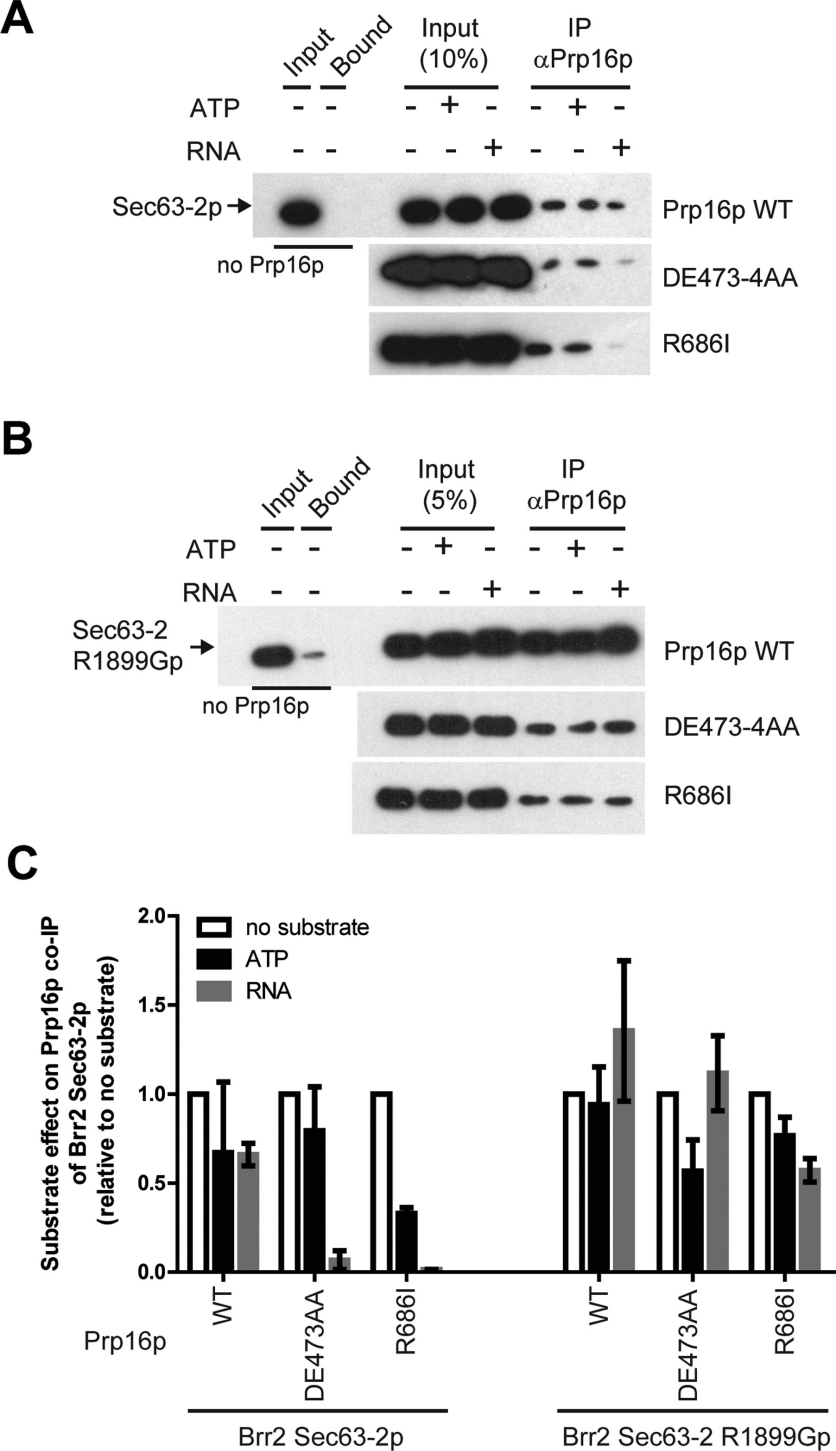
The direct interaction of Brr2p with Prp16p *in vitro* is modulated by RNA. (**A** and **B**) Co-immunoprecipitation of WT Sec63-2p (A) or of mutant Sec63-2 R1899Gp (B) by WT or mutant Prp16p in presence or absence of ATP (5 mM) or poly I,C RNA (500 ng/μl) or, as a control, in absence of Prp16p. Sec63-2p was detected using anti-His-HRP conjugated antibodies (Santa Cruz). (**C**) Quantitation of the co-immunoprecipitation efficiencies from three independent experiments performed as in panels A and B. Error bars represent the standard error to the mean.

In order to test for direct physical interaction of the Brr2 Sec63-2p domain with Prp16p, we performed immunoprecipitations with recombinant proteins purified from bacterial cells. The Brr2 H2-Sec63-2 fragment was only soluble in high salt buffers that were unsuitable for our *in vitro* assays (data not shown). We therefore purified recombinant Brr2 Sec63-2p, Sec63-*2* R1899Gp (other mutant versions of Sec63-2 protein were largely insoluble), Prp16p and several mutant versions of Prp16p. WT and two mutant Prp16 proteins (DE473-4AA and R686I) were immunoprecipitated with similar efficiencies with an anti-Prp16p antibody (Supplementary Figure S4A), and they efficiently co-immunoprecipitated Brr2 Sec63-2p irrespective of RNase A treatment (Supplementary Figure S4A and B), indicating that the interaction is direct and not mediated by contaminating RNA. The interaction is also largely unaffected by an excess of ATP (Figure 4A). To test the effect of saturating amounts of RNA we used poly I,C RNA, because it contains a mix of RNA fragments of various sizes, and RNA helicases generally show no RNA specificity *in vitro* (3,36). The presence of RNA may cause a mild reduction of the interaction of Sec63-2p with WT Prp16p (Figure 4A and C), but more strongly reduced the interaction with the Prp16 mutant proteins DE473-4AA and R686I. In contrast, there was little or no effect of ATP or RNA on the interaction of Sec63-2 R1899Gp with Prp16p WT and mutant proteins (Figure 4B and C).

### Brr2 Sec63-2p interferes with the RNA binding and ATPase activities of Prp16p

**Figure 5.**
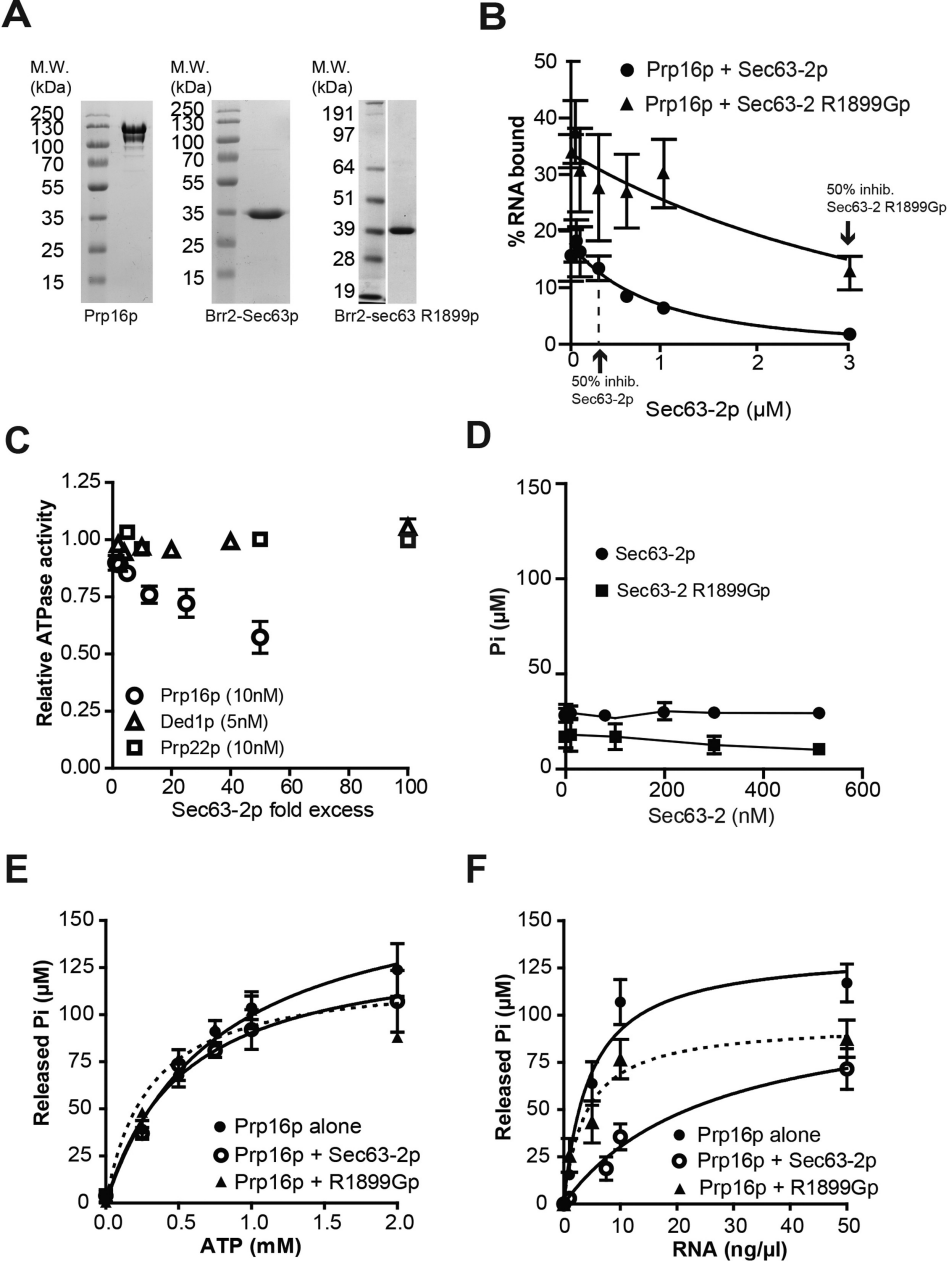
Sec63-2p reduces the RNA binding and ATPase activities of Prp16p *in vitro.* (**A**) Recombinant Prp16p, Brr2 Sec63-2p and Brr2 Sec63-2 R1899Gp. SDS Page gel containing 3 μg of proteins, stained with Coomassie blue. Molecular weight markers are indicated on the left. (**B**) Quantitation of EMSA assessing Prp16p (300 nM) binding to single stranded RNA (2 nM) in the presence of Sec63-2p (black circles) or Sec63-*2* R1899Gp (black triangles). Error bars represent the standard error to the mean from 2 (Sec63-2p) and 3 (Sec63-2 R1899Gp) independent experiments. (**C**) Relative ATPase activities of Prp16p (10 nM, open circles), Prp22p (10 nM, open squares) and Ded1p (5 nM, open triangles) in presence of increasing amounts of Sec63-2p (represented as molar excess to the helicases), and in presence of 50-ng/μl total yeast RNA. **D**. Effect of Sec63-2p (black circles) and Sec63-2 R1899Gp (black squares) on the ATPase activity of Prp16p (10 nM) in the absence of RNA. (**E**) ATP dependence of Prp16p (10 nM) ATPase activity in absence (black circles) or presence (open circles) of 500-nM Sec63-2p or of Sec63-2 R1899Gp (black triangle and dashed line), and in presence of 50 ng/μl total yeast RNA. (**F**) RNA dependence of Prp16p (10 nM) ATPase activity in presence (open circles) or absence (black circles) of 500-nM Sec63-2p or Sec63-2 R1899Gp (black triangle and dashed line) and 1-mM ATP. The value for RNA-independent ATP hydrolysis was deduced prior to analysis.

To better understand the relationship between RNA, Prp16p and the Brr2p Sec63-2 domain, we performed gel shift assays, using purified recombinant proteins (Figure 5A) and a 44-base ssRNA that was bound efficiently by Prp16p *in vitro* but to which the Sec63-2 WT and R1899G proteins bound very poorly (Supplementary Figures S5A and B). Selecting a concentration of Prp16p (300 nM) that bound between 30 and 40% of the RNA substrate, we added increasing amounts of WT Sec63-2p, which greatly reduced the association of Prp16p with the RNA (*K*_i,app_ = 479 ± 2 nM, Figure 5B and Supplementary Figure S5C). Addition of Sec63-2 R1899G mutant protein also reduced RNA binding to Prp16p, although the effect was much weaker (*K*_i,app_ = 3097 ± 3 nM, Figure 5B and Supplementary Figure S5D). Importantly, at the concentration of Sec63-2p and sec63-2 R1899Gp that reduce Prp16p RNA binding by 50%, Sec63-2p and Sec63-2 R1899Gp bind <5% and ∼10% of the RNA respectively (Supplementary Figure S5). Together with the co-immunoprecipitation results, this suggests a competition between Sec63-2p and RNA for binding to Prp16p.

Measuring the ATPase activity of Prp16p *in vitro*, we observed a 2-fold reduction in the presence of excess Sec63-2p, whereas there was no effect of Sec63-2p on the ATPase activities of Ded1p (a DEAD-box RNA helicase involved in translation (38,39)) or Prp22p (Prp22p does not interact with Brr2p C-terminus in Y2H assay; data not shown) even at 100- or 50-fold molar excess respectively (Figure 5C). As previously observed by Schwer and Guthrie (33), Prp16p had a very low RNA-independent ATPase activity. This activity was unaffected by Sec63-2p (Figure 5D). In the presence of Sec63-2p at 500 nM, the maximal inhibition of Prp16p was not yet reached (Figure 5C). Therefore, the kinetic parameters measured represent apparent values (Table 2). Sec63-2p increased the *K*_Mapp,RNA_ of Prp16p 6-fold, without altering the *K*_M,ATP_ (Table 2). This indicates that the presence of WT Sec63-2p reduces the capacity of RNA to bind and stimulate ATP hydrolysis but that it has no effect on the capacity of Prp16p to bind ATP. Sec63-2 R1899G mutant protein also inhibited the RNA-dependent activity of Prp16p, though to a lesser extent and, like the WT protein, it showed little or no effect on the RNA-independent activity (Figure 5D). Sec63-2 R1899Gp had little effect on the *K*_Mapp,RNA_ of Prp16p but reduced the *K*_Mapp,ATP_ about 2-fold (Figure 5E and F and Table 2) suggesting that binding of the Sec63 mutant protein affects the conformation of the ATP binding pocket. The *k*_cat_ of the Prp16p ATPase activity which measures the velocity of the hydrolysis step remained unchanged in the presence of WT or mutant Sec63-2p (Table 2) indicating that Sec63-2p did not impair the catalytic step.

**Table 2.** Apparent Michaelis-Menten parameters of ATPase activities

	*K*_Mapp,ATP_ (μM)	*K*_Mapp,RNA_ (ng/μl)^†^	*k*_cat,app_ (min^−1^)	*V*_max_ (μM/min)
Prp16p	751 ± 181	4.6 ± 1.4 (13.6 ± 4.2 μM)	183 ± 41	3.0 ± 0.3
Prp16p + Sec63-2p	534 ± 178	27.1 ± 12.4 (79.9 ± 36.4 μM)	154 ± 19	2.5 ± 0.5
Prp16p + Sec63-2 R1899Gp	297 ± 101	3.9 ± 1.6 (11.5 ± 4.8 μM)	135 ± 14	2.1 ± 0.1

To characterize further the functional relationship between RNA and Sec63-2p, we measured the ATPase activity of Prp16p in the presence of increasing amounts of Sec63-2p and of RNA (Supplementary Figure S6A). Significantly, *K*_Mapp,RNA_ increased as the concentration of Sec63-2p in the reaction increased, while it remained stable in the presence of increasing amounts of Sec63-2 R1899Gp (up to 500 nM; Supplementary Figures S6B and C), confirming the inhibitory effect of Sec63-2p on ATP hydrolysis by Prp16p, and a lack of this effect with the Brr2-R1899G mutant protein.

## DISCUSSION

Activities of splicing RNA helicases must be finely controlled in order to ensure timely rearrangements of RNA–RNA and RNA–protein interactions during the splicing cycle (2,3,40). For example, the activity of Brr2p can be modulated by the U5 snRNP proteins Prp8p and Snu114p (7,9,12,14,15) as well as by its own C-terminus (18). Here, we demonstrate allele-specific genetic and physical interactions between Brr2p and Prp16p that support a close functional relationship between these two helicases.

Using mutagenesis and a Y2H screen we uncovered determinants in the Brr2p C-terminus that are important for its interaction with the Prp16 helicase, and identified the first known mutants of the second helicase module of Brr2p that cause a splicing defect. Importantly, mutations that were identified based on Y2H interactions also affect the genetic interaction with *PRP16* when present in full-length *BRR2*, validating this focused genetic approach. However, as discussed below, an increased or decreased Y2H interaction does not predict a corresponding effect on splicing activity. The various genetic and physical interactions observed between the *brr2* and *prp16* alleles are summarized in Table 3.

**Table 3.**
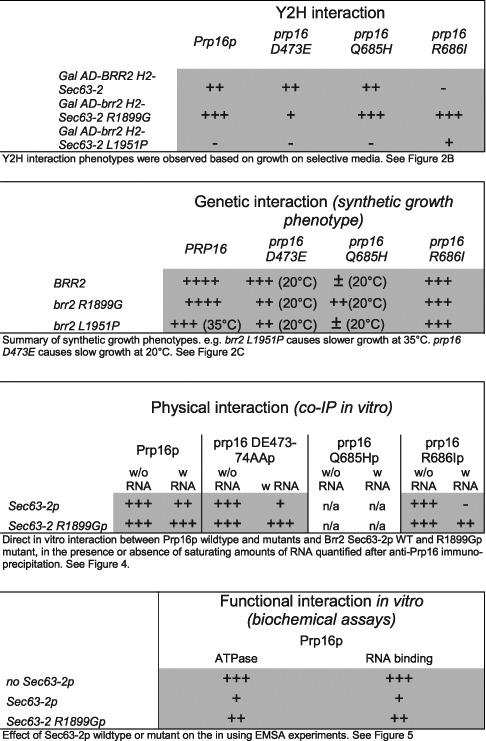
Summary of experimental results

We show that Brr2p C-terminal fragments can inhibit the RNA binding and RNA stimulation of the ATPase activity of Prp16p *in vitro*. In addition, the observation that RNA inhibits interaction of the Prp16 R686I mutant protein with recombinant WT Sec63-2p argues for a competition between RNA and Sec63-2p for interaction with Prp16p (but not necessarily for the same binding site in Prp16p). The strong genetic interactions observed between *brr2* alleles and *prp16* motif VI alleles (*prp16-Q685H, prp16-R686I, prp16-R686Q*) suggest that modulation of Prp16p activity by Brr2p occurs at the level of RNA stimulation of the ATPase activity, a known function of motif VI in RNA helicases of the DExD/H-box families (4). Upon binding of the RNA cofactor the active site of DExD/H-box RNA helicases is remodeled, allowing the formation of an ATP-binding pocket competent for hydrolysis (3). In DEAD-box RNA helicases, the first arginine of motif VI (equivalent to R686 in Prp16p) can interact with a conserved phenylalanine from the RNA binding motif IV (41). This interaction may be important for transmitting the signal to the ATP-binding pocket that RNA has been bound, which triggers the formation of a catalytically competent active site. In the DEAH-box splicing factor Prp43p, the motif IV phenylalanine and the motif VI arginine are in very close proximity (42,43). Also, in Mg-ADP bound Prp43p, Q421 (equivalent to Q685 in Prp16p), interacts with R425 of motif VI, which is necessary for ATP hydrolysis. By analogy, mutation of R686I in Prp16p might disrupt transmission of the RNA binding signal, whereas the Q685H mutation may alter the stimulation of ATP hydrolysis.

This could explain the suppression by *brr2-R1899G* of the growth defect of *prp16-Q685H*. By allowing RNA more access to the active site of Prp16p, *brr2-R1899G* may stimulate defective ATPase activity caused by *prp16-Q685H.* In contrast, if the *prp16-R686I* mutation prevents recognition of the RNA-bound state, increasing access of RNA to the mutant protein will likely not suppress the defect, and no suppression of the growth defect was observed.

Analysis of splicing by RT-qPCR showed a low level of *ACT1* pre-mRNA accumulation and no lariat accumulation with the *brr2*-*R1899G* and *brr2*-*L1951P* mutations. The step 1 splicing defect may be due to an effect of the *brr2* mutations on Brr2p function or, more likely (as the mutated residues lie in a region of Brr2p that does not interact with the Brr2p N-terminal helicase module; Figure S2A) on Prp2p activity prior to step 1. Somewhat surprisingly, the *prp16* alleles, *prp16-Q685H, prp16-R686I* and *prp16-R686Q*, displayed only very mild splicing defects for *ACT1, DBP2* and *RPL28* transcripts that are insufficient to explain the severe growth defect. These *prp16* alleles carry mutations in motif VI that has been proposed to affect RNA stimulation of the ATPase activity of this family of RNA helicases. Koodathingal *et al*. (44) presented evidence for a role for Prp16 in proofreading 5′ splice site cleavage. This suggests a mechanism whereby mutations in *prp16* that affect RNA stimulation of ATP hydrolysis might give rise to a splicing defect that specifically affects suboptimal introns. A more in-depth analysis, such as RNA-seq, might identify transcripts that are more strongly affected by this *prp16* allele.

The GIM screen identified a negative genetic interaction between *brr2-L1951* and *isy1Δ*, which itself suppresses the *prp16-302* defect (32). However, *brr2-L1951P* does not suppress *prp16-302*, indicating that the *brr2* and *isy1Δ* mutations have distinct effects on Prp16p. *ISY1* deletion seems to facilitate release of the cold-sensitive ATPase-defective Prp16-302p from spliceosomes by an unknown mechanism (32), possibly affecting the conformation of the spliceosome but not necessarily by enhancing the ATPase activity of Prp16p. Our model suggests that mutant Brr2 proteins that have weaker interactions with Prp16p (as for Brr2-L1951p) may fail to prevent RNA binding to Prp16p, resulting in stimulation of its ATPase activity, leading to premature release of Prp16p from the spliceosome. Therefore, *brr2-L1951P* and *isy1Δ* may destabilize interaction of WT Prp16p with spliceosomes by different mechanisms which, when combined, have an additive effect. This does not mean that the *brr2-L1951P* mutation should necessarily suppress the same *prp16* alleles as deletion of *ISY1.*

**Figure 6.**
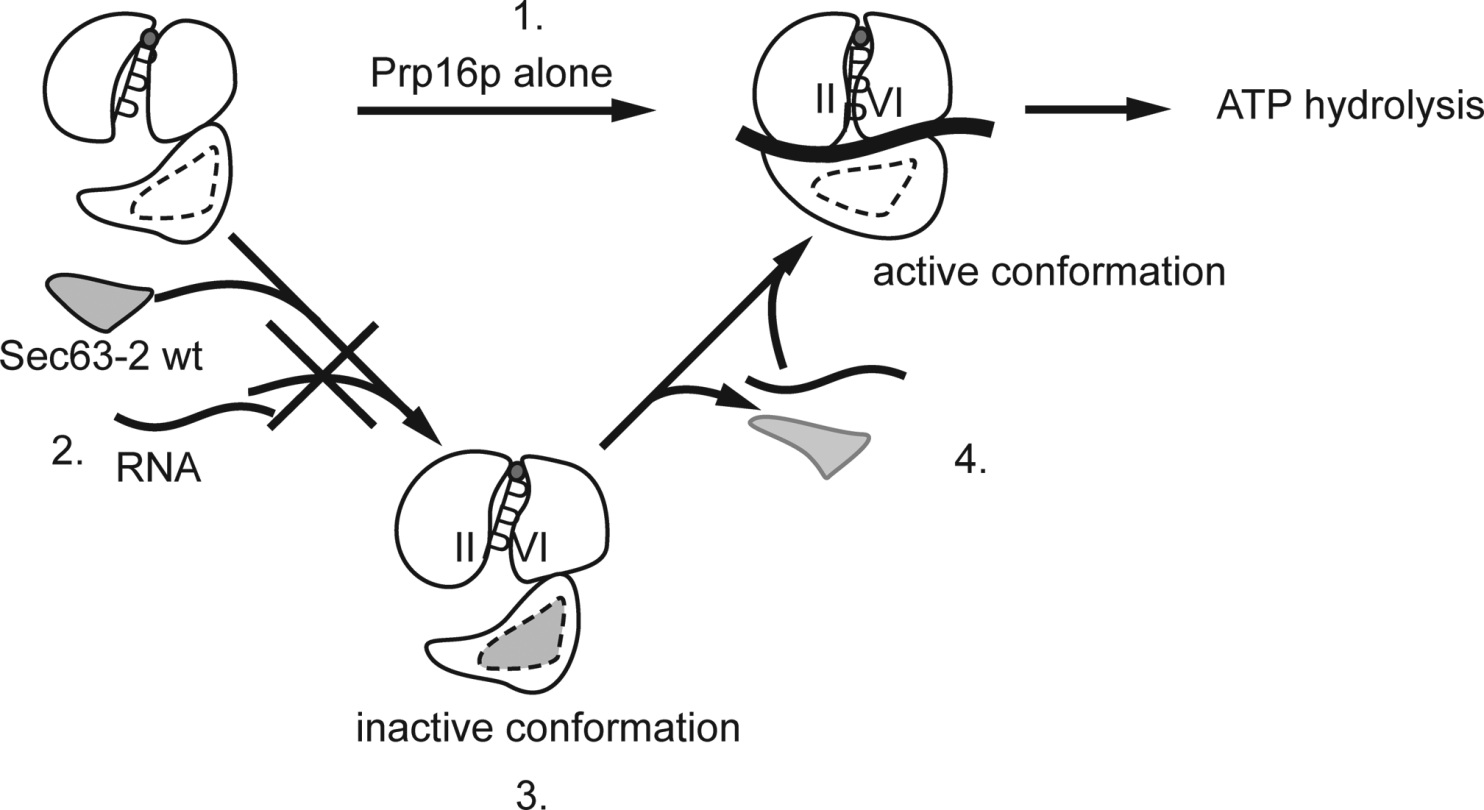
Model for modulation of Prp16p ATPase activity by Brr2 Sec63-2p. In the absence of Brr2p C-terminus, Prp16p binds its two substrates (ATP and RNA) and as a consequence, hydrolyzes ATP (1). In the presence of Brr2p C-terminus, binding of the Sec63-2 domain to Prp16p allosterically prevents RNA from accessing the RNA binding site (2), thereby inhibiting ATP hydrolysis (3). Upon release of Brr2p C-terminus, RNA can access the RNA binding site of Prp16p (4) and promote the conformational changes that lead to ATP hydrolysis.

How might Brr2 affect RNA binding to Prp16p? The crystal structure of the DEAH-box RNA helicase Prp43p reveals the presence of a 5′ hairpin (5′HP) and an OB-fold domain that are conserved among DEAH-box helicases (30,42,43). In the ADP-bound crystal of Prp43p, the 5′HP interacts with the OB-fold domain and together they occlude the RNA binding cleft. We propose a model in which Brr2p Sec63-2 domain acts as a ‘dimmer’ switch for Prp16p activity, possibly by locking the 5′HP-OB-fold interaction to exclude RNA from the active site (Figure 6). Conceivably, mutants of Brr2p in the Sec63-2 domain (such as *brr2-R1899G*) might indirectly alter the position of the 5′HP and/or the OB-fold domain of Prp16p, allowing RNA to bind and stimulate ATP hydrolysis, albeit less efficiently than in the absence of Sec63-2p.

The inhibition by Sec63-2p of the ATPase activity of Prp16p but not of Prp22p, suggests that Brr2 Sec63-2p interaction with Prp16p is mediated by specific structures in the DEAH-box proteins. Although the C-termini of DEAH-box proteins share high similarity, they also present clear differences (30) that could be responsible for the modulation of RNA binding/unwinding and protein–protein interaction (3,29). We have identified a region (from R1899 to L1951) in the second Sec63 domain of Brr2p, including helices α3, α4 and α5 (the ‘ratchet helix’; Supplementary Figure S2A) (17,19) that appears to be of major importance for the interaction with Prp16p and Prp2p *in vivo.* With the WT Brr2p, it is possible that modifying the position of these structures relative to each other, e.g. upon protein–protein interaction, post-translational modification or ATP binding to Brr2p H2 domain, impacts on the overall folding of the Brr2p C-terminus, thereby altering its specificity for interacting partners, such as Prp2p or Prp16p, in the spliceosome. This suggests a very complex interplay between RNA helicases in the splicesome's catalytic center, with Brr2p playing a pivotal role that is very difficult to dissect *in vivo* and extremely challenging to analyze *in vitro.*

## SUPPLEMENTARY DATA

Supplementary Data are available at NAR Online.

SUPPLEMENTARY DATA

## References

[1] Will C.L., Luhrmann R. (2011). Spliceosome structure and function. Cold Spring Harb. Perspect. Biol..

[2] Cordin O., Beggs J.D. (2013). RNA helicases in splicing. RNA Biol..

[3] Cordin O., Hahn D., Beggs J.D. (2012). Structure, function and regulation of spliceosomal RNA helicases. Curr. Opin. Cell Biol..

[4] Tanner N.K., Linder P. (2001). DExD/H box RNA helicases: from generic motors to specific dissociation functions. Mol. Cell.

[5] Raghunathan P.L., Guthrie C. (1998). RNA unwinding in U4/U6 snRNPs requires ATP hydrolysis and the DEIH-box splicing factor Brr2. Curr. Biol..

[6] Hahn D., Kudla G., Tollervey D., Beggs J.D. (2012). Brr2p-mediated conformational rearrangements in the spliceosome during activation and substrate repositioning. Genes Dev..

[7] Small E.C., Leggett S.R., Winans A.A., Staley J.P. (2006). The EF-G-like GTPase Snu114p regulates spliceosome dynamics mediated by Brr2p, a DExD/H box ATPase. Mol. Cell.

[8] Fourmann J.B., Schmitzova J., Christian H., Urlaub H., Ficner R., Boon K.L., Fabrizio P., Luhrmann R. (2013). Dissection of the factor requirements for spliceosome disassembly and the elucidation of its dissociation products using a purified splicing system. Genes Dev..

[9] Mozaffari-Jovin S., Wandersleben T., Santos K.F., Will C.L., Lührmann R., Wahl M.C. (2014). Novel regulatory principles of the spliceosomal Brr2 RNA helicase and links to retinal disease in humans. RNA Biol..

[10] Laggerbauer B., Achsel T., Luhrmann R. (1998). The human U5–200kD DEXH-box protein unwinds U4/U6 RNA duplices in vitro. Proc. Natl. Acad. Sci. U.S.A..

[11] Hacker I., Sander B., Golas M.M., Wolf E., Karagoz E., Kastner B., Stark H., Fabrizio P., Luhrmann R. (2008). Localization of Prp8, Brr2, Snu114 and U4/U6 proteins in the yeast tri-snRNP by electron microscopy. Nat. Struct. Mol. Biol..

[12] Bellare P., Small E.C., Huang X., Wohlschlegel J.A., Staley J.P., Sontheimer E.J. (2008). A role for ubiquitin in the spliceosome assembly pathway. Nat. Struct. Mol. Biol..

[13] Maeder C., Kutach A.K., Guthrie C. (2009). ATP-dependent unwinding of U4/U6 snRNAs by the Brr2 helicase requires the C terminus of Prp8. Nat. Struct. Mol. Biol..

[14] Mozaffari-Jovin S., Santos K.F., Hsiao H.H., Will C.L., Urlaub H., Wahl M.C., Luhrmann R. (2012). The Prp8 RNase H-like domain inhibits Brr2-mediated U4/U6 snRNA unwinding by blocking Brr2 loading onto the U4 snRNA. Genes Dev..

[15] Mozaffari-Jovin S., Wandersleben T., Santos K.F., Will C.L., Luhrmann R., Wahl M.C. (2013). Inhibition of RNA helicase Brr2 by the C-terminal tail of the spliceosomal protein Prp8. Science.

[16] Nguyen T.H.D., Li J., Galej W.P., Oshikane H., Newman A.J., Nagai K. (2013). Structural basis of Brr2-Prp8 interactions and implications for U5 snRNP biogenesis and the spliceosome active site. Structure.

[17] Pena V., Jovin S.M., Fabrizio P., Orlowski J., Bujnicki J.M., Luhrmann R., Wahl M.C. (2009). Common design principles in the spliceosomal RNA helicase Brr2 and in the Hel308 DNA helicase. Mol. Cell.

[18] Santos K.F., Jovin S.M., Weber G., Pena V., Luhrmann R., Wahl M.C. (2012). Structural basis for functional cooperation between tandem helicase cassettes in Brr2-mediated remodeling of the spliceosome. Proc. Natl. Acad. Sci. U.S.A..

[19] Zhang L., Xu T., Maeder C., Bud L.O., Shanks J., Nix J., Guthrie C., Pleiss J.A., Zhao R. (2009). Structural evidence for consecutive Hel308-like modules in the spliceosomal ATPase Brr2. Nat. Struct. Mol. Biol..

[20] Kim D.H., Rossi J.J. (1999). The first ATPase domain of the yeast 246-kDa protein is required for in vivo unwinding of the U4/U6 duplex. RNA.

[21] van Nues R.W., Beggs J.D. (2001). Functional contacts with a range of splicing proteins suggest a central role for Brr2p in the dynamic control of the order of events in spliceosomes of Saccharomyces cerevisiae. Genetics.

[22] Chen H.C., Tseng C.K., Tsai R.T., Chung C.S., Cheng S.C. (2013). Link of NTR-Mediated Spliceosome Disassembly with DEAH-Box ATPases Prp2, Prp16, and Prp22. Mol. Cell. Biol..

[23] Liu H.L., Cheng S.C. (2012). The interaction of Prp2 with a defined region of the intron is required for the first splicing reaction. Mol. Cell. Biol..

[24] Fromont-Racine M., Rain J.C., Legrain P. (1997). Toward a functional analysis of the yeast genome through exhaustive two-hybrid screens. Nat. Genet..

[25] Longtine M.S., McKenzie A., Demarini D.J., Shah N.G., Wach A., Brachat A., Philippsen P., Pringle J.R. (1998). Additional modules for versatile and economical PCR-based gene deletion and modification in Saccharomyces cerevisiae. Yeast.

[26] Boeke J.D., LaCroute F., Fink G.R. (1984). A positive selection for mutants lacking orotidine-5′-phosphate decarboxylase activity in yeast: 5-fluoro-orotic acid resistance. Mol. Gen. Genet..

[27] Janke C., Magiera M.M., Rathfelder N., Taxis C., Reber S., Maekawa H., Moreno-Borchart A., Doenges G., Schwob E., Schiebel E. (2004). A versatile toolbox for PCR-based tagging of yeast genes: new fluorescent proteins, more markers and promoter substitution cassettes. Yeast.

[28] Alexander R.D., Innocente S.A., Barrass J.D., Beggs J.D. (2010). Splicing-dependent RNA polymerase pausing in yeast. Mol. Cell.

[29] Cordin O., Tanner N.K., Doere M., Linder P., Banroques J. (2004). The newly discovered Q motif of DEAD-box RNA helicases regulates RNA-binding and helicase activity. EMBO J..

[30] Kudlinzki D., Schmitt A., Christian H., Ficner R. (2012). Structural analysis of the C-terminal domain of the spliceosomal helicase Prp22. Biol. Chem..

[31] Decourty L., Saveanu C., Zemam K., Hantraye F., Frachon E., Rousselle J.C., Fromont-Racine M., Jacquier A. (2008). Linking functionally related genes by sensitive and quantitative characterization of genetic interaction profiles. Proc. Natl. Acad. Sci. U.S.A..

[32] Villa T., Guthrie C. (2005). The Isy1p component of the NineTeen complex interacts with the ATPase Prp16p to regulate the fidelity of pre-mRNA splicing. Genes Dev..

[33] Schwer B., Guthrie C. (1992). A dominant negative mutation in a spliceosomal ATPase affects ATP hydrolysis but not binding to the spliceosome. Mol. Cell. Biol..

[34] Hotz H.R., Schwer B. (1998). Mutational analysis of the yeast DEAH-box splicing factor Prp16. Genetics.

[35] Schwer B., Guthrie C. (1991). PRP16 is an RNA-dependent ATPase that interacts transiently with the spliceosome. Nature.

[36] Pyle A.M. (2011). RNA helicases and remodeling proteins. Curr. Opin. Chem. Biol..

[37] Madhani H.D., Guthrie C. (1994). Genetic interactions between the yeast RNA helicase homolog Prp16 and spliceosomal snRNAs identify candidate ligands for the Prp16 RNA-dependent ATPase. Genetics.

[38] Del Campo M., Mohr S., Jiang Y., Jia H., Jankowsky E., Lambowitz A.M. (2009). Unwinding by local strand separation is critical for the function of DEAD-box proteins as RNA chaperones. J. Mol. Biol..

[39] Tarn W.Y., Chang T.H. (2009). The current understanding of Ded1p/DDX3 homologs from yeast to human. RNA Biol..

[40] Wahl M.C., Will C.L., Luhrmann R. (2009). The spliceosome: design principles of a dynamic RNP machine. Cell.

[41] Banroques J., Cordin O., Doere M., Linder P., Tanner N.K. (2008). A conserved phenylalanine of motif IV in superfamily 2 helicases is required for cooperative, ATP-dependent binding of RNA substrates in DEAD-box proteins. Mol. Cell. Biol..

[42] He Y., Andersen G.R., Nielsen K.H. (2010). Structural basis for the function of DEAH helicases. EMBO Rep..

[43] Walbott H., Mouffok S., Capeyrou R., Lebaron S., Humbert O., van Tilbeurgh H., Henry Y., Leulliot N. (2010). Prp43p contains a processive helicase structural architecture with a specific regulatory domain. EMBO J..

[44] Koodathingal P., Novak T., Piccirilli J.A., Staley J.P. (2010). The DEAH box ATPases Prp16 and Prp43 cooperate to proofread 5′ splice site cleavage during Pre-mRNA splicing. Mol. Cell.

