# Supplementary material for: Brr2p carboxy-terminal Sec63 domain modulates Prp16 splicing RNA helicase
Source: Nucleic Acids Res. 2014 Nov 26;42(22):13897–910. doi: 10.1093/nar/gku1238 (PMC4267655; doi:10.1093/nar/gku1238)
Supplement: SUPPLEMENTARY DATA [file supp_42_22_13897__index.html]

Brr2p carboxy-terminal Sec63 domain modulates Prp16 splicing RNA helicase — Brr2p carboxy-terminal Sec63 domain modulates Prp16 splicing RNA helicase — SUPPLEMENTARY DATA 

# Brr2p carboxy-terminal Sec63 domain modulates Prp16 splicing RNA helicase

## SUPPLEMENTARY DATA

**Files in this Data Supplement:**

- SUPPLEMENTARY DATA
