# Supplementary material for: Brr2p carboxy-terminal Sec63 domain modulates Prp16 splicing RNA helicase
Source: Nucleic Acids Res. 2014 Nov 26;42(22):13897–910. doi: 10.1093/nar/gku1238 (PMC4267655; doi:10.1093/nar/gku1238)
Supplement: SUPPLEMENTARY DATA [file supp_gku1238_nar-03661-a-2013-File008.pdf]

SUPPLEMENTARY INFORMATION

Brr2p carboxy-terminal Sec63 domain modulates the Prp16 splicing RNA helicase

Olivier Cordin[1,2,#,] Daniela Hahn[1], Ross Alexander[1,3], Amit Gautam[1,4], Cosmin Saveanu[5] , J. David Barrass[1] and Jean D. Beggs[1,#]

[1] Wellcome Trust Centre for Cell Biology, University of Edinburgh, King's Buildings, Mayfield Road, Edinburgh, EH9 3JR, UK

[2] Current address: IBPC, CNRS FRE 3630, 13, rue Pierre & Marie Curie , 75005, Paris, France

[3] Current address: Crop and Soil Systems , SRUC, King's Buildings, West Mains Road, Edinburgh EH9 3JG, UK

[4] Current address: Department of Medicine, Imperial College London, London W12 0NN, UK

[5] Institut Pasteur, Génétique des Interactions Macromoléculaires, Center National de la Recherche Scientifique, Paris, France

#To whom correspondence should be addressed
JDB: Tel: +44-131-650-5351; Fax: +44-131-650-5351; e-mail: jbeggs@ed.ac.uk
Correspondence may also be addressed to:
OC: Tel: +33-1-57-27-66-69; e-mail: olivier.cordin@univ-paris-diderot.fr

CONTENTS:

SUPPLEMENTARY TABLES S1 to S4

SUPPLEMENTARY FIGURES S1 to S6

SUPPLEMENTARY MATERIALS AND METHODS

SUPPLEMENTARY REFERENCES

SUPPLEMENTARY TABLES

Supplementary Table S1

Plasmids used in this study

Plasmid	source
**Yeast Two-Hybrid plasmids**	
pACTIIstop	Fromont-Racine *et al*., 1997
pACTIIstop-H2	this study
pACTIIstop-Sec63-2	this study
pACTIIstop-H2-Sec63-2	this study
pACTIIstop-H2-Sec63-2 library and plasmids isolated (Table 1)	this study
pBTM116	Fromont-Racine *et al*., 1997
pBTM116-PRP2	this study
pBTM116-PRP16	this study
pBTM116-PRP22	this study
pBTM116-PRP43	this study
pBTM116-prp16-L335F	this study
pBTM116-prp16-K379R	this study
pBTM116-prp16-D473E	this study
pBTM116-prp16-H476D	this study
pBTM116-prp16-T507A	this study
pBTM116-prp16-Q685H	this study
pBTM116-prp16-R686Q	this study
pBTM116-prp16-R686I	this study
pBTM116-prp16-302	this study
**Shuffle plasmids**	
pRS316-BRR2	this study
pRS315-BRR2	this study
pRS315-brr2-R1107P	this study
pRS315-brr2-R1899G	this study
pRS315-brr2-K1925R0	this study
pRS315-brr2-L1883P	this study
pRS315-brr2-L1930P	this study
pRS315-brr2-A1932P	this study
pRS315-brr2-L1951P	this study
pRS315-brr2-I2071T	this study
pRS315-brr2-I2073N	this study
pRS315-brr2-S2148P	this study
pRS316-BRR2/PRP16	this study
pRS314-PRP16	this study
pRS314-prp16-D473E	this study
pRS314-prp16-Q685H	this study
pRS314-prp16-R686I	this study
***E. coli* expression plasmids**	
pDONR223 (entry vector)	Invitrogen
pET22b-PRP16-His	P. Fabrizio
pET22b-prp16-K379A-His	this study
pET22b-prp16-DE473-4AA- His	this study
pET22b-prp16-R686I- His	this study
pETM11-His-Sec2	V. Pena
pETM11-His-Sec63-2 R1899G	this study
pET21a-PRP2-His	P. Fabrizio

Supplementary Table S2
Yeast strains used in this study

Strain	genotype
L40ΔG	*MAT**a** his3Δ200 trp1-901 leu2-3*, *122 ade2 LYS2*:: (*4lexAop-HIS3*) *URA3*::(*lexAop-lacZ*) Δ*gal4::KAN*
W303	*MAT**a** leu2-3,112 trp1-1 can1-100 ura3-1 ade2-1 his3-11,15*
W303 *brr2*Δ	W303 *MAT**a*** Δ*brr2*::*KanMX6* pRS316-*BRR2*
W303 *brr2*Δ*/isy1*Δ	W303 *MAT**a*** Δ*brr2*::*KanMX6* Δisy1::NatNT2 pRS316-*BRR2*
W303 *brr2*Δ*/prp16*Δ	W303 *MAT**a*** Δbrr2::*KanMX6* Δ*prp16*::*NatNT2* pRS316-*BRR2/PRP16*

Supplementary Table S3
List of oligonucleotides used for RT-qPCR experiments

Name	sequence
ACT1 (Lariat + Lariat-Exon2) F	AGGGGCTTGAAATTTGGAAAAA
ACT1 (Lariat + Lariat-Exon2) R	GCAAGCGCTAGAACATACATAGTACA
ACT1 (Pre-mRNA) F	AGGGGCTTGAAATTTGGAAAAA
ACT1 (Pre-mRNA) R	GCAACAAAAAGAATGAAGCAATCG
ACT1 (Pre-mRNA + Lariat-Exon2) F	TTGCTTCATTCTTTTTGTTGCT
ACT1 (Pre-mRNA + Lariat-Exon2) R	GCAAAACCGGCTTTACACAT
ACT1 (mRNA) F	TCGAAAATTTACTGAATTAACAATGGA
ACT1 (mRNA) R	GCAAAACCGGCTTTACACAT
ACT1 (Exon2) F	GCTGCTTTGGTTATTGATAACGGTTC
ACT1 (Exon2) R	GATGGGAAGACAGCACGAGGAG

Supplementary Table S4

Genes conferring synthetic growth defects when combined with *brr2-L1930P* or *brr2-L1951P* in the GIM screen.

ORF	Gene name	*Fitness brr2* L1930P	*Fitness brr2* L1951P
YCL029C	BIK1	-0.073	0.013
YPR057W	BRR1	-0.090	-0.021
YPL064C	CWC27	-0.053	0.011
YNL001W	DOM34	-0.059	0.005
YIL103W	DPH1	-0.079	0.000
YLR172C	DPH5	-0.079	0.000
YBR065C	ECM2	-0.085	-0.004
YGL020C	GET1	-0.109	0.032
YHL031C	GOS1	-0.070	0.026
YIL041W	GVP36	-0.112	-0.031
YGR187C	HGH1	-0.057	0.006
YMR186W	HSC82	-0.164	-0.003
YNR032C-A	HUB1	-0.074	-0.015
YDR332W	IRC3	-0.057	-0.068
YJR050W	ISY1	-0.243	-0.106
YJR097W	JJJ3	-0.054	0.009
YNL238W	KEX2	-0.082	-0.054
YOR322C	LDB19	-0.048	-0.117
YHR156C	LIN1	-0.312	-0.171
YDR378C	LSM6	-0.136	-0.010
YML062C	MFT1	-0.069	-0.014
YJL123C	MTC1	-0.073	-0.036
YIL007C	NAS2	-0.033	-0.075
YBR188C	NTC20	-0.070	-0.014
YGR178C	PBP1	-0.081	0.030
YLR016C	PML1	-0.152	-0.037
YNL082W	PMS1	-0.064	-0.033
YHR157W	REC104	-0.190	-0.091
YDR137W	RGP1	-0.053	0.057
YOL143C	RIB4	-0.007	-0.073
YER056C-A	RPL34A	-0.051	0.003
YLR268W	SEC22	-0.129	-0.027
YDR363W-A	SEM1	-0.095	0.000
YKR029C	SET3	-0.053	-0.009
YMR216C	SKY1	-0.080	-0.005
YGL115W	SNF4	-0.057	0.009
YOR308C	SNU66	-0.274	-0.133
YKR031C	SPO14	-0.051	-0.032
YOR027W	STI1	-0.097	0.027
YGR129W	SYF2	-0.071	0.008
YHR025W	THR1	-0.055	-0.001
YDR457W	TOM1	-0.022	-0.090
YMR022W	UBC7	-0.055	0.009
YNL229C	URE2	-0.003	-0.132
YJR049C	UTR1	-0.054	-0.056
YLR386W	VAC14	-0.063	0.004

YDR431W	YDR431W	-0.072	0.029
YGR079W	YGR079W	-0.079	-0.003
YHR130C	YHR130C	-0.054	-0.021
YKR040C	YKR040C	-0.069	-0.003
YKR041W	YKR041W	-0.098	-0.035
YNL140C	YNL140C	-0.103	0.028
YPL068C	YPL068C	0.013	-0.056

Shaded lines indicate genes whose products are involved in pre-mRNA splicing.

SUPPLEMENTARY FIGURES

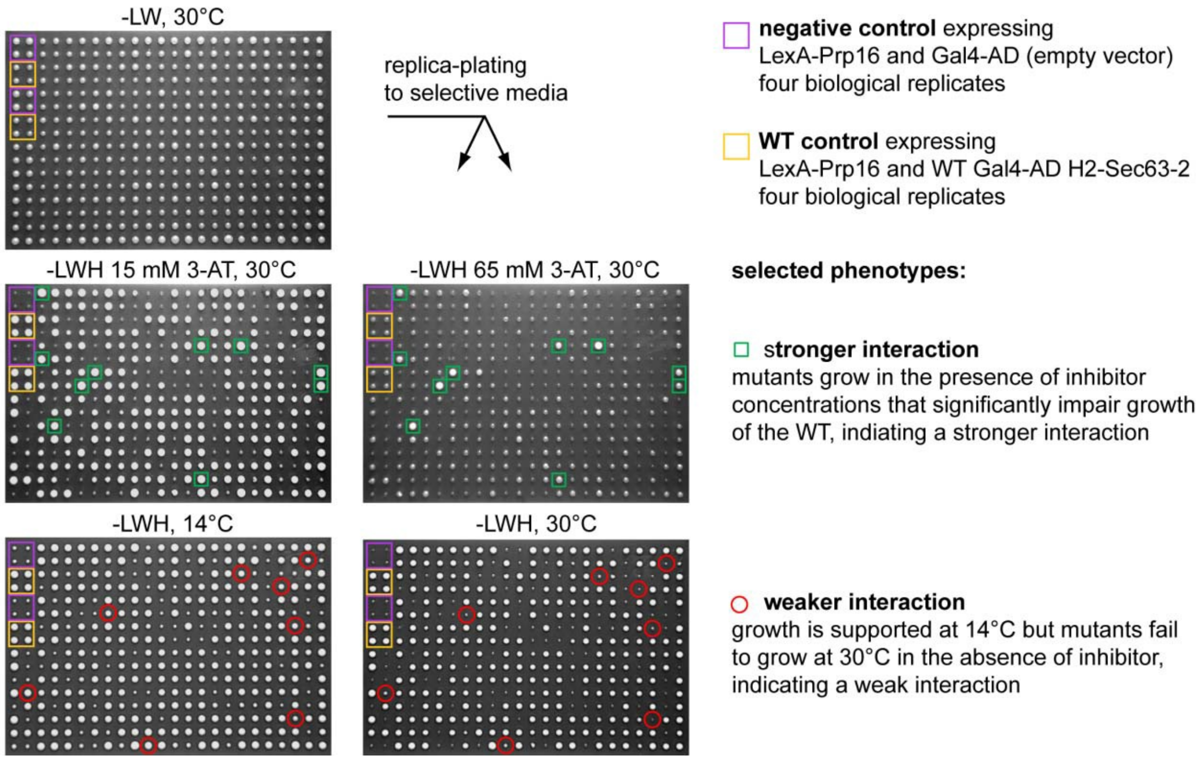

Supplementary Figure S1. Yeast-two hybrid screen to isolate *brr2-Sec63-2* mutants that have altered interaction with Prp2p and/or Prp16p.

Two mutant phenotypes were selected based on their growth properties on selective media. Screens with Prp16 and Prp2 baits were carried out consecutively (the images shown serve as an example). A library of *brr2-H2-Sec63-2* two-hybrid constructs, randomly mutated across the Sec63-2 domain was co-transformed with the bait plasmid. Transformants were spotted to a master-plate, then replica-plated onto selective media. Mutations with stronger Y2H interactions (in green squares) exhibit resistance to higher concentrations of 3-AT (up to 65 mM for Prp16p; up to 20 mM for Prp2p) than the WT. Mutations causing weaker Y2H interactions (circled in red) display a loss of growth in the absence of histidine at 30°C but not at 14°C (most also show reduced or no resistance to 3-AT).

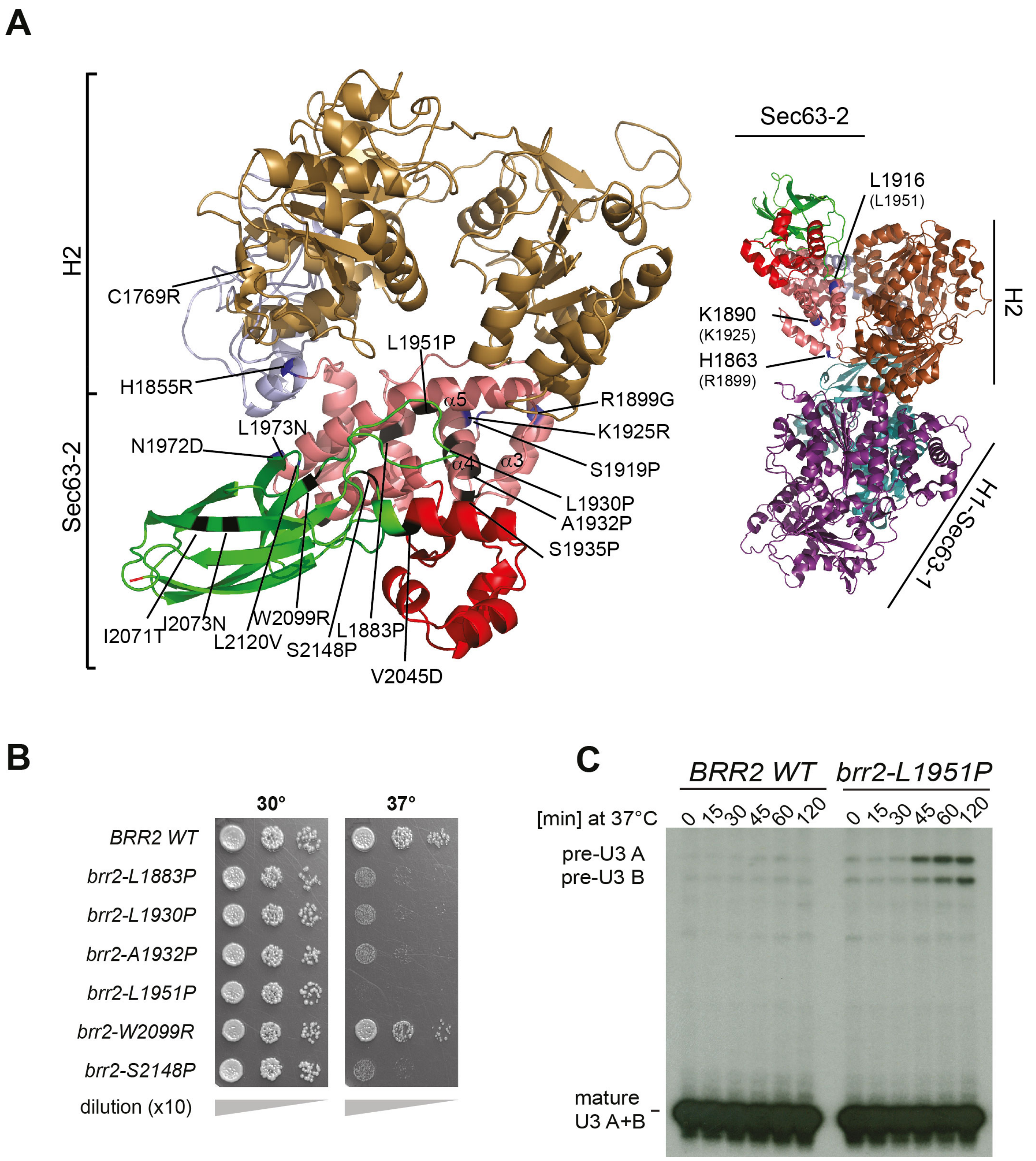

Supplementary Figure S2. Sec63-2 mutations in the structure of Brr2, and in vivo phenotypes.

A. Left: Location of the Sec63-2 mutations within the structure of the carboxy-terminal helicase module of Brr2 (based on human Brr2 structure, PDB: 4F91). Structure was drawn using PyMol software. Helicase domain H2 is colored in brown, the WH2 domain is grey, the amino-terminal helical domain of Sec63-2 is pink, the middle helical domain is red and the fibronectin-3 like domain is green. Positions of single mutations isolated in the screens are highlighted in black (weaker interaction) or blue (stronger interaction). Right: position of H1863 (R1899 in yeast), K1890 (K1925 in yeast) and L1916 (L1951 in yeast) in full-length human Brr2 structure. Selected residues are colored in dark blue. Structural domains are

colored as before except Brr2 H1 domain, which is colored in purple, and Sec63-1 in light blue.
B. Heat sensitivity of various *brr2* alleles that were isolated in the Y2H screen as weaker interactors of Prp16p. Following 5-FOA selection, cultures were serially diluted, spotted to YPDA and grown at 30°C or 37°C.
C. Primer extension analysis of U3 snoRNA from WT *BRR2* and *brr2-L1951P* strains. Cultures in mid-log phase were shifted to non-permissive condition (37°C) for the indicated times. Accumulation of pre-U3A and B indicates a splicing defect *in vivo*.

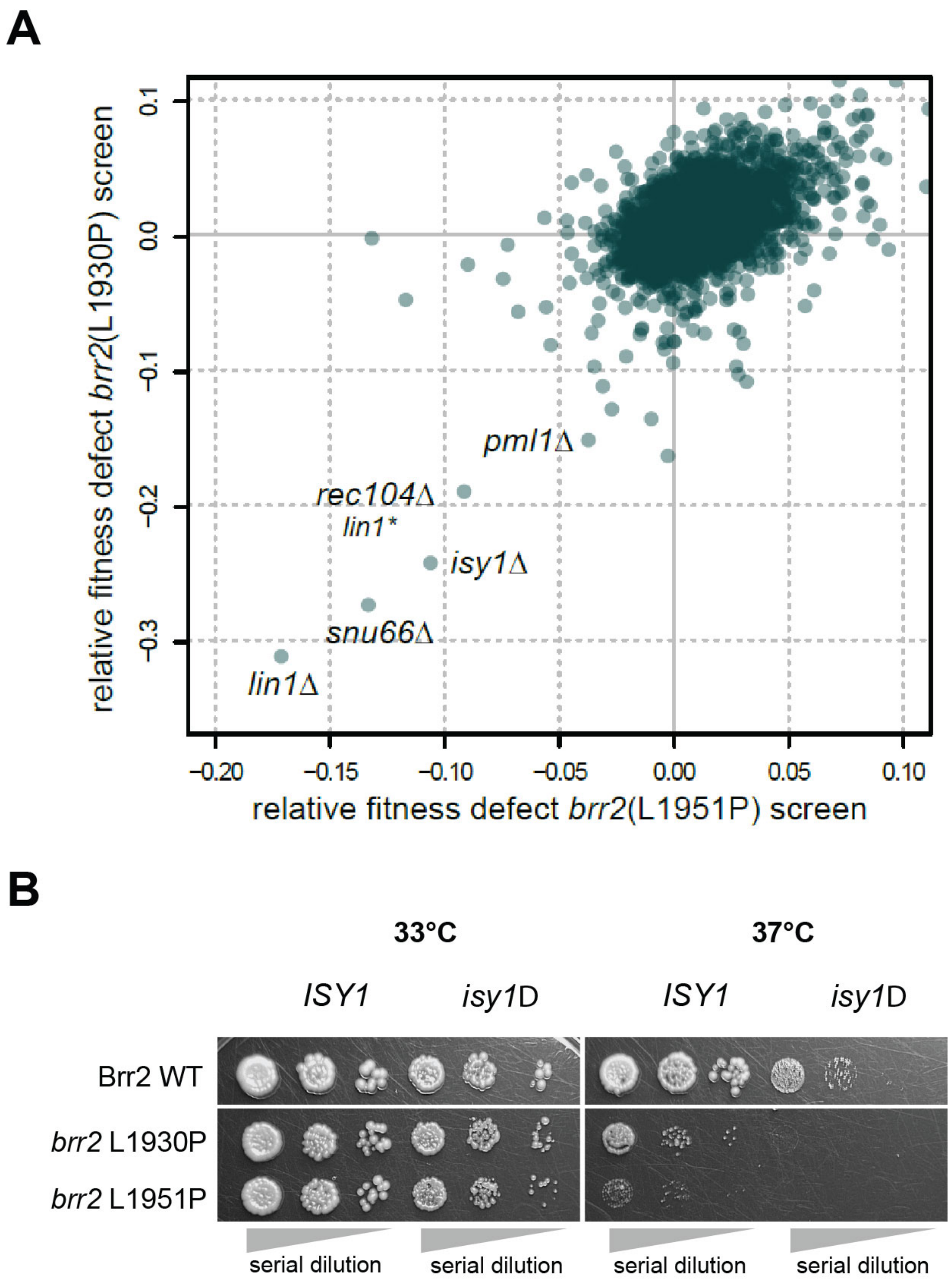

Supplementary Figure S3. *Brr2 Sec63-2* mutant alleles interact genetically with the NTC factor *ISY1*.

A. GIM screen identifies genetic interaction between two *brr2-Sec63-2* mutant alleles and several splicing factors. Sensitive and quantitative genetic interaction mapping screens were carried out at 30°C, according to (Decourty et al. 2008), with the *brr2-L1930P* or *brr2-L1951P* allele as bait. The relative growth rates of double-deletion strains are represented as a scatter plot, in which mutations that caused synthetic growth inhibition in combination with both *brr2* alleles lie on the diagonal in the lower-left quadrant. Only *LIN1* and *SNU66,* which encode protein components of the U5 snRNP and U4/U6.U5 snRNP complex respectively (and therefore are in close physical association with Brr2p), showed a stronger genetic interaction than did *ISY1*.

B. Deletion of *ISY1* exacerbates phenotypes of *brr2* mutants. The slow-growth defect of *brr2* mutants with substitutions in Sec63 -2 combined with *isy1Δ* was confirmed on YPDA plates and increased heat sensitivity was observed at 33°C and 37°C.

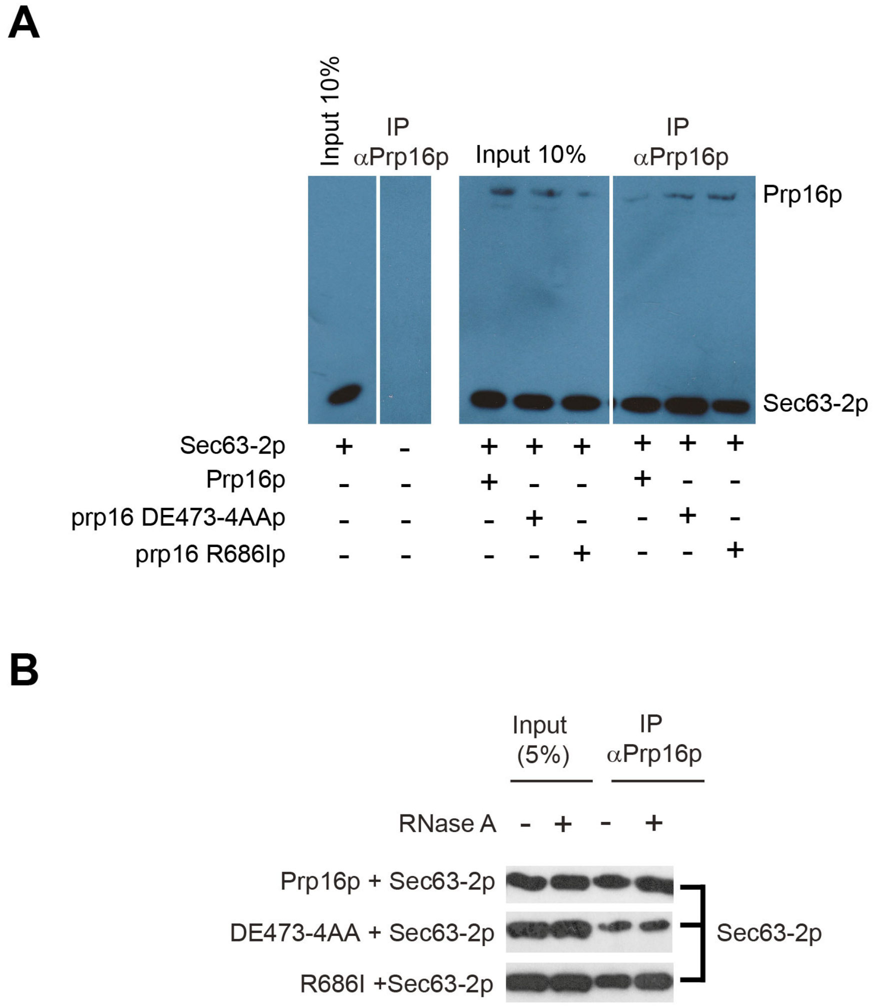

Supplementary Figure S4. Brr2 Sec63-2p interaction with Prp16p is not RNA mediated.
A. Sec63-2p is efficiently co-immunoprecipitated with wild-type or mutant Prp16p.
B. Anti-Prp16 co-immunoprecipitation of Sec63-2p with wild-type or mutant Prp16p with or without RNase A (30ng/µl) treatment.
In both panels A and B, bands are detected using anti-His-HRP conjugated antibodies (Santa Cruz) to detect the His-tagged recombinant proteins.

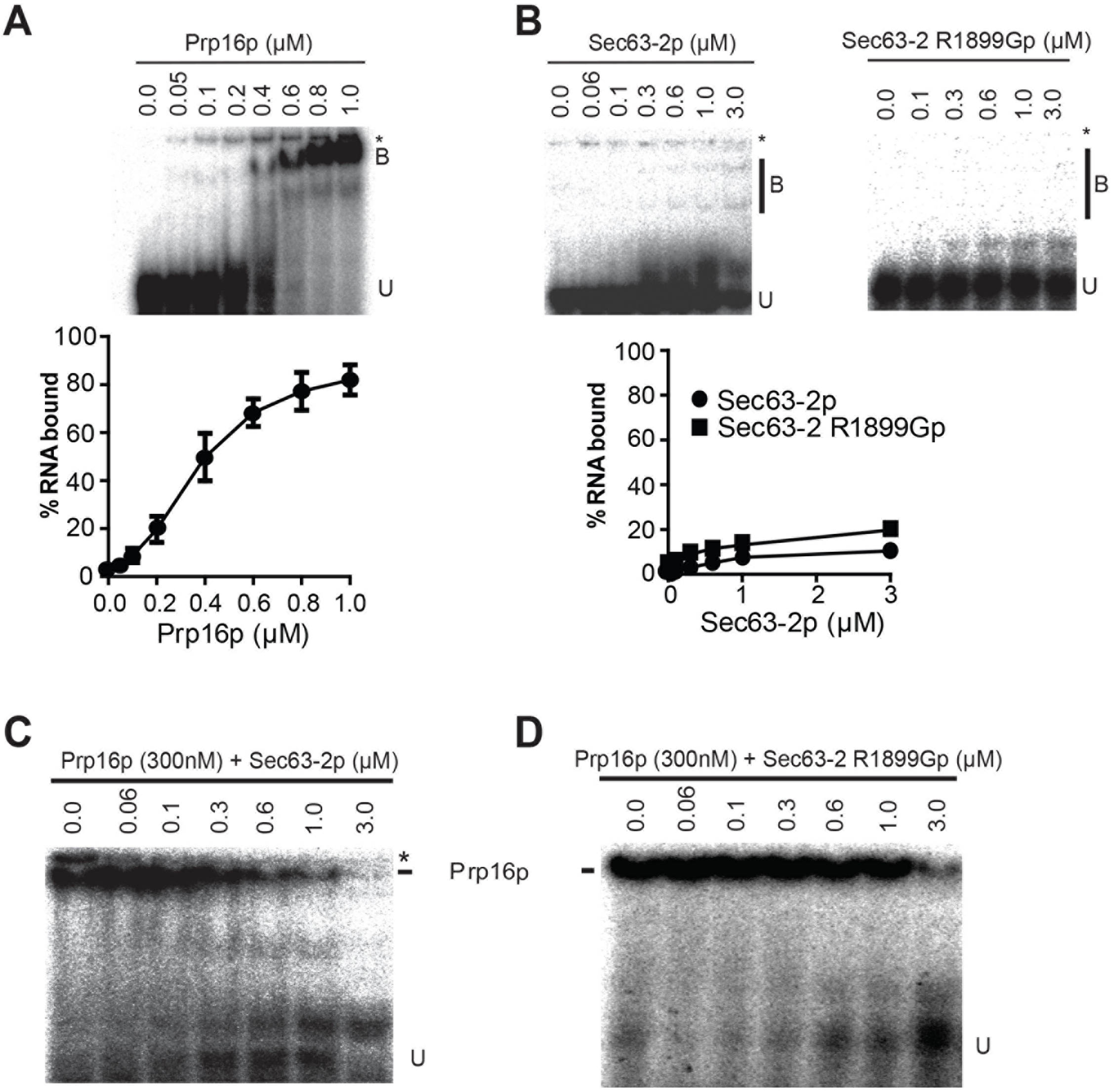

Supplementary Figure S5. ***In vitro*** **RNA binding by Prp16p, Brr2 Sec63-2p and Brr2 Sec63-2 R1899Gp and competitive binding assays.**

A. EMSA of 2nM of RNA in the presence of increasing concentrations of Prp16p. The proportion of Prp16p-bound RNA ((bound)x100/total lane) from 3 independent experiments is shown in the graph below. Error bars represent the SEM.
B. EMSA of 2nM of RNA in the presence of increasing concentrations of Brr2 Sec63-2p or Sec63-2 R1899Gp. The proportion of Sec63-2p-bound RNA ((bound)x100/total lane) from 3 independent experiments is shown in the graph below. Error bars represent the SEM.
C. Representative EMSA of single-stranded RNA in the presence of 300nM Prp16p and increasing amounts of Sec63-2p. Prp16p-bound RNA is indicated beside the gel; the star indicates the origin of migration, U indicates the unbound RNA.
D. EMSA of single-stranded RNA in the presence of 300nM Prp16p and increasing amounts of Sec63-2 R1899Gp.

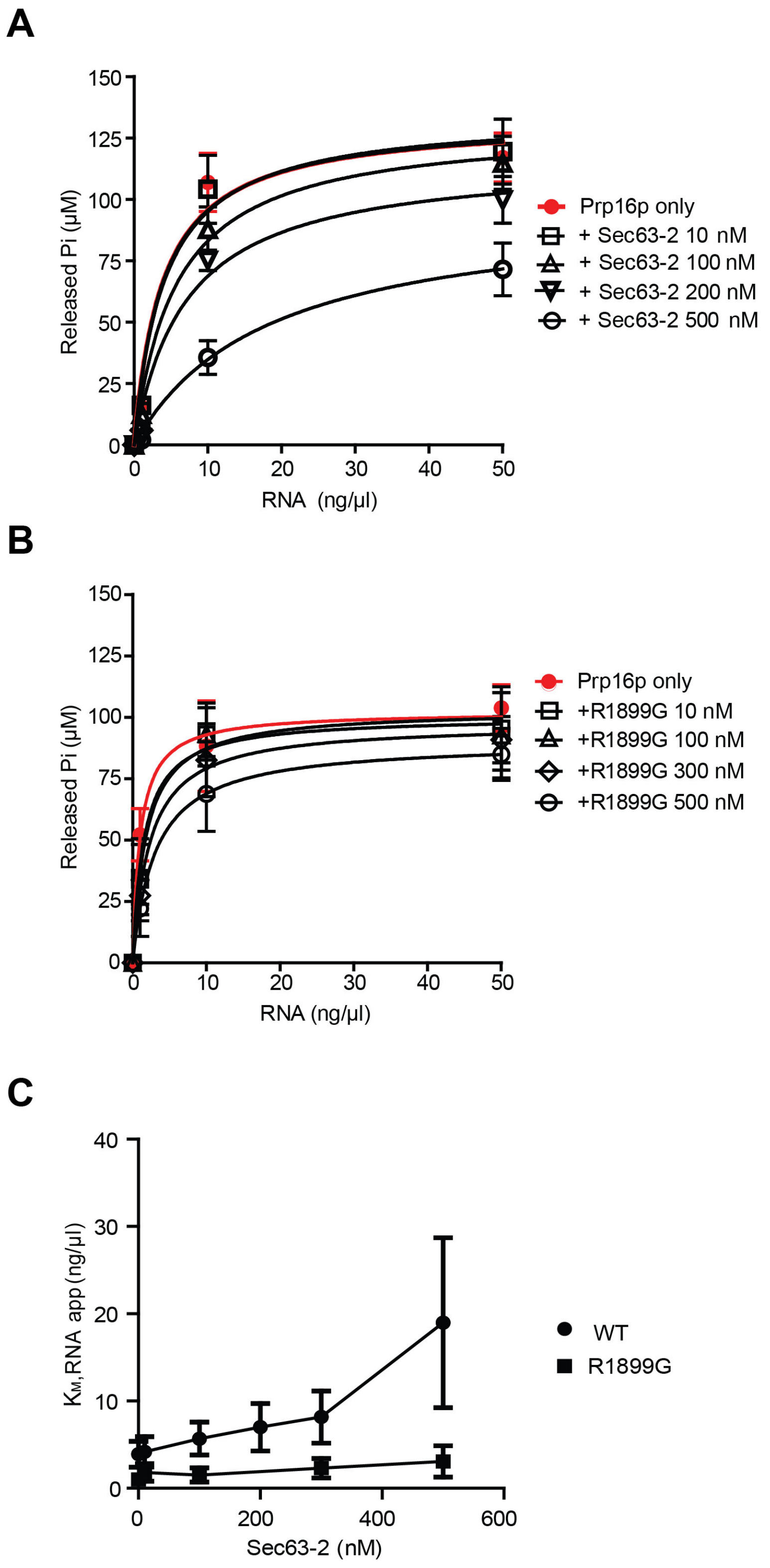

Supplementary Figure S6.

Supplementary Figure S6. RNA dependence of Prp16p ATPase activity in the presence of increasing concentrations of Brr2 Sec63-2p or Brr2 Sec63-2 R1899Gp.

A. RNA dependence of Prp16p ATPase activity in the presence of increasing concentrations of Brr2 Sec63-2p. The graph shows the mean of 3 independent experiments. Error bars represent the SEM.
B. RNA dependence of Prp16p ATPase activity in the presence of increasing concentrations of Brr2 Sec63-2 R1899Gp. The graph shows the mean of 3 independent experiments. Error bars represent the SEM.
For A. and B., to focus on the effect of Brr2 Sec63-2 R1899Gp on the RNA-dependent ATPase activity of Prp16p, RNA-independent background activity was subtracted prior analysis of the data.
C. Evolution of the apparent $K_{M,RNA}$ in the presence of increasing concentrations of Brr2 Sec63-2p or Sec63-*2* R1899Gp. Values are derived from the experiments shown in A and B.

SUPPLEMENTARY MATERIALS AND METHODS

Cloning procedures

Brr2p domains H2-Sec63-2 and Sec63-2 were PCR-amplified and cloned into pBlueScript using *SpeI* and *XhoI* restriction sites. Brr2 H2 was PCR amplified from pBS-H2-Sec63-2. Subcloning in pACTII-stop was done by amplifying the coding sequence of *BRR2* domains from pBS vectors using oligonucleotides carrying the desired restriction sites.
LexA-bait fusions with the ORFs of *PRP2*, *PRP16*, *PRP22* and *PRP43* were generated by InFusion cloning (Takara). Gal4AD-prey fusions for H2-Sec63-2, H2 and Sec63-2 were constructed in pACTII-stop by standard cloning using *XmaI* / *BamHI* restriction sites. Mutations in *PRP2* and *PRP16* were introduced following the Quick Change protocol (Promega).

Plasmid shuffle and growth assay

A W303 *brr2*Δ strain was constructed by replacing one chromosomal copy of *BRR2* with the kanMX6 cassette in W303 2n (1). The heterozygous deletion was confirmed by Colony PCR. The *URA3* marked plasmid pRS316-*BRR2* was introduced and *brr2*Δ haploids (W303 *brr2*Δ) were isolated after sporulation and tetrad dissection. A plasmid -shuffling approach was used to observe the phenotypes of brr2 mutants (2). The wild-type *BRR2* gene including -276 nt 5' flanking sequence and +229 nt 3' flanking sequence was PCR amplified and cloned into a *SacI* / *XmaI* digested pRS315 backbone to create pRS315-*BRR2*. Mutant derivatives of pRS315-*BRR2* were generated by site-directed mutagenesis (Quick Change, Promega). Plasmids used are listed in Table S1. For growth assays overnight cultures of 5-FOA selected transformants were spotted on YPDA agar. Plates were incubated for 2 days at 25°, 30°, 37° or for 5 days at 18° or 15°.
The *ISY1* ORF was replaced with the natNT2 cassette (3) in W303 *brr2*Δ to construct W303 *brr2*Δ/*isy1*Δ.
W303 *brr2*Δ/*prp16*Δ was constructed by replacing one genomic copy of *PRP16* with the natNT2 cassette in a diploid, heterozygous *brr2*Δ background. Growth was supported by the *URA3* marked plasmid pRS316-*BRR2*/*PRP16*, expressing Brr2p and Prp16p. After sporulation and tetrad dissection *brr2*Δ/*prp16*Δ haploids were selected (W303 *brr2*Δ/*prp16*Δ). pRS314-*PRP16* was generated by cloning the *PRP16* ORF including -304 nt 5' flanking sequence and +170 nt 3' flanking sequence into the *XmaI* site of pRS314. Mutant derivatives were constructed by site-directed mutagenesis. W303 *brr2*Δ/*prp16*Δ was co-transformed with pRS314-*PRP16* and pRS315-*BRR2* or mutant versions thereof for plasmid shuffle assays.
Growth curves were obtained using Sunrise shakers/readers (Tecan). Shortly, overnight cultures (2ml) of W303 *brr2Δ/prp16Δ* transformed with pRS314-*PRP16* or mutants and

pRS315-*BRR2* or mutants were diluted in the wells of a 96-well plate (flat bottom) to an OD of 0.05 in 100μl of -L-W media and grown at 25°C for 4h. Cultures were then transferred into new incubators set at 20°C, 25°C, 35°C or 37°C. OD600nm was measured automatically every 15 minutes for a period of 24 or 48h. Each culture was duplicated in a single plate and two plates were grown at each temperature in two independent incubators.

Primer extension
After 5-FOA selection, W303 *brr2*Δ + pRS315-*BRR2* (or *brr2*) were grown for the indicated time at 37°C and RNA was extracted. Primer U3 exon 2, 5' CCAAGTTGGATTCAGTGGCTC (4) was ^{32}P-end labelled and used for reverse transcription of 2 μg total RNA. Products were analysed on 7% polyacrylamide gels and signals detected by autoradiography.

SUPPLEMENTARY REFERENCES

1. Longtine, M.S., McKenzie, A., 3rd, Demarini, D.J., Shah, N.G., Wach, A., Brachat, A., Philippsen, P. and Pringle, J.R. (1998) Additional modules for versatile and economical PCR-based gene deletion and modification in Saccharomyces cerevisiae. *Yeast*, **14**, 953-961.
2. Boeke, J.D., LaCroute, F. and Fink, G.R. (1984) A positive selection for mutants lacking orotidine-5'-phosphate decarboxylase activity in yeast: 5-fluoro-orotic acid resistance. *Mol Gen Genet*, **197**, 345-346.
3. Janke, C., Magiera, M.M., Rathfelder, N., Taxis, C., Reber, S., Maekawa, H., Moreno-Borchart, A., Doenges, G., Schwob, E., Schiebel, E. *et al.* (2004) A versatile toolbox for PCR-based tagging of yeast genes: new fluorescent proteins, more markers and promoter substitution cassettes. *Yeast*, **21**, 947-962.
4. Fabrizio, P., Laggerbauer, B., Lauber, J., Lane, W.S. and Luhrmann, R. (1997) An evolutionarily conserved U5 snRNP-specific protein is a GTP-binding factor closely related to the ribosomal translocase EF-2. *Embo J*, **16**, 4092-4106.
